# Everyday challenges facing high-risk older people living in the community: a community-based participatory study

**DOI:** 10.1186/s12877-020-1470-y

**Published:** 2020-02-17

**Authors:** Tsuyoshi Okamura, Chiaki Ura, Mika Sugiyama, Madoka Ogawa, Hiroki Inagaki, Fumiko Miyamae, Ayako Edahiro, Yukiko Kugimiya, Mutsuko Okamura, Mari Yamashita, Shuichi Awata

**Affiliations:** Tokyo Metropolitan Institute of Gerontology, 35-2 Sakae-cho, Itabashi-ku, Tokyo, 173-0015 Japan

**Keywords:** Dementia, Cognitive impairment, Community care, Social support

## Abstract

**Background:**

Considering the real-world experiences of those with cognitive impairments is important in building a positive community for older people. Community-based participatory research is an important methodology for investigators focused on improving community health. The aim of this study was to 1) investigate factors associated with the continuation of community dwelling among high-risk older people and 2) to create a model of an inclusive community space for older people in the largest housing complex district in Tokyo.

**Methods:**

From 198 residents who completed all three steps (mail, face-to-face, and home-visit) of a previous large-scale epidemiological survey, we identified 66 residents who were at high-risk of moving out of the community. These participants underwent 6 months of regular assessments by experienced researchers to identify the factors associated with continuing to live in the community.

We also employed a community action approach to develop a community space for residents in the study district where more than two researchers who were medical professionals served as staff. The services offered by the space were continuously improved according to user feedback. The function of this center was evaluated during interdisciplinary research meetings.

**Results:**

After 6 months, among the 66 high-risk residents, 49 people were living in the community and 12 people had moved out of the community. Those who could not continue to live in the community had greater unmet needs in terms of social support, especially daily living support and housing support. In addition, their families perceived a heavier burden of care. Interestingly, dementia diagnosis via the DSM-5, clinical dementia rating, physical health, mental health, and long-term care usage did not predict the outcome.

Through discussions with guests, we equipped the space with various services such as coordination of community care and networking with existing organizations.

**Conclusions:**

Merely providing healthcare and long-term care might not be sufficient to support community living in people with cognitive impairments. Daily living support and housing support should be provided in the context of a broad health services package. For this purpose, creating a comfortable community space for residents and community workers is essential.

## Background

The number of people who are 65 years and older in Japan exceeded 28% of the total population in 2018 [1]. Japan is becoming the most aged society in the world [2, 3]. As the society ages, the number of people with dementia is expected to grow to around 10,000,000 by the mid-century [4], exceeding 10% of the total population. The Japanese government has created a national dementia strategy with the goal to reform the care system from one that is institution-based to one that is community-based. This “community-based integrated care system” would enable older people to age in the community for as long as possible [5].

Aging and the associated changes can threaten one’s emotional, social, physical, and financial well-being if an individual is not able to successfully adapt to these changes. Poor adaptation to changes, as well as increasing need for care, can lead individuals to lose function such that they may no longer be able to live in the community. According to Golant [6], the promotion of environments in which residential normalcy is a common goal can prevent unproductive discussions regarding age-segregated vs. age-integrated environments. Residential normalcy is not a static state, but exists with a dynamic tension that modulates according to residential comfort and residential mastery. To deliver residential normalcy for older people with cognitive impairments, real-world evidence concerning the experiences of community-dwelling older people with cognitive impairment is essential, which have not been fully explored [7].

Previous studies have mainly focused on the survival rate of people with dementia. For instance, Ohara [8] reported that the 5-year survival rate of dementia patients in Japan improved from 47.3 to 65.2% between 1988 and 2002. Prospective studies conducted in France [9] and the UK [10] both estimated the survival time from disease onset to be 4.5 years. Further, van de Vorst [11] reported a linkage between low socioeconomic status (SES) and mortality in dementia patients. Specifically, the 5-year mortality rate in men was 74% among those in the lowest SES tertile and 57% among those in the highest. Similarly, Chen [12] reported a higher mortality rate associated with living in a rural area (adjusted hazard ratio = 2.96) and having depression (adjusted hazard ratio = 4.15). However, few studies have gathered information about the experiences of people with dementia living in the community. In addition, the specific variables that impact community living are still unclear. From a clinical viewpoint, the factors that influence one’s ability to continue living in the community could be related to dementia, mental health, physical health, the characteristics of the community, socioeconomic status, and the need or lack of social support, in addition to sociodemographic variables.

According to Grill and Galvin [13], only a small portion of community dwelling older people participate in clinical trials and biomarker studies, and participants in such trials tend to be younger, more educated, and are more likely to be Caucasian. To address this, Grill and Galvin recommended the use of community-based participatory research principles. Similarly, Lepore et al. [14] reported that people with dementia were frequently excluded from research, and encouraged investigators to involve these individuals as participants in studies about care and service.

In conventional public health research, participants are usually assessed at multiple points in time. In such studies, follow-up assessments are a major challenge. For instance, if a participant was no longer registered in a particular area, privacy protection in Japan [15] can make it difficult to judge whether the participant has moved to another area or has been institutionalized/ hospitalized. In addition, the experiences of the participants are often unclear. One approach for investigating individual experience is qualitative studies. However, the participants in such in-depth studies are not necessarily a representative sample. To address this, in the present study, we used a representative sampling method.

The aim of this study was 1) to investigate factors associated with the continuation of community dwelling amongst older individuals with cognitive impairment and 2) to create a model of an inclusive community space in the center of the largest housing complex district in Tokyo, which is known to have weakened social ties and an aging resident population. This study was based on the community-based participatory research (CBPR) approach [16, 17]. In CBPR, researchers and community stakeholders form equitable partnerships and co-construct research for the mutual and complementary goals of community health improvement and knowledge production.

## Methods

### Theoretical framework

According to the CBPR framework, we used two methods (Table 1). The first employed a high-risk approach for identifying challenges within the community. We conducted a survey with three steps: assessments via a large epidemiological survey and selection of 66 high-risk participants (quantitative), follow-up assessments (qualitative), and re-assessment after 6 months (quantitative).

**Table 1:** Rationale and purpose of the study methods according to community-based participatory research principles

Community-based participatory research	
High-risk approach	At the start of the study, we included all of the older people in the community. We considered that this would decrease the chance of bias in our study, and improve the chance that the city office would be interested in our research. We conducted follow-up assessments with the participants who were judged to be high-risk. The residents regarded this to be ethical. During the follow-up period, the high-risk participants had opportunities to interact with researchers and individuals or organizations in the community, which we anticipated would enhance partnership building.
Community action approach	Instead of basing our study around an institution or hospital, we built a community space in the field, with the goal of developing a dementia-friendly community with a human-rights based approach.

The second used a community action approach to develop partnerships with the residents. We built a community space with the goal of creating a dementia-friendly community (DFC). DFCs are becoming more numerous globally and are a common goal amongst people with dementia, their families, policymakers, and researchers [18, 19]. We also adapted a human-rights-based approach [18, 20]. As a strategy to embed a human-rights-based approach in our project, we adapted the PANEL framework proposed by the Alzheimer Scotland organization. PANEL stands for Participation, Accountability, Non-discrimination, Empowerment, and Legality.

**Fig. 1:**
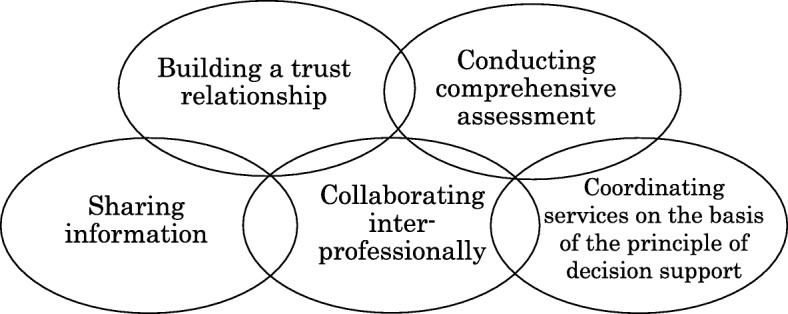
Coordinating service providers to meet the needs of service users and ensure that they receive integrated, person-centered social support. To apply this approach to persons with dementia, we emphasized five processes in a repeated and parallel manner: building a trust relationship, conducting comprehensive assessments, sharing information, collaborating inter-professionally, and coordinating services on the basis of the principle of decision support

**Fig. 2:**
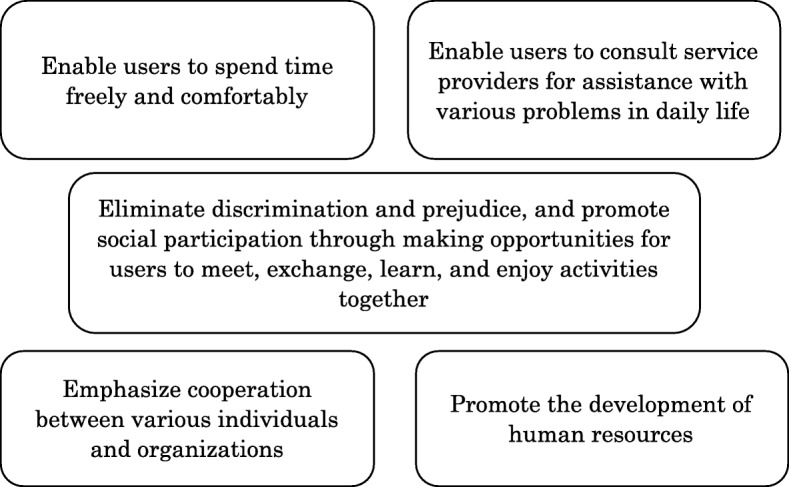
Networking refers to building a social network, which is a structure that enables individuals to continuously provide and receive social support. Promoting mutual social support is a key factor in enabling a person with dementia to live in a community with hope and dignity. To effectively enhance social support, we recommend that community spaces 1) enable users to spend time freely and comfortably, 2) enable users to consult service providers for assistance with various problems in daily life, 3) eliminate discrimination and prejudice, and promote social participation through making opportunities for users to meet, exchange, learn, and enjoy activities together, 4) emphasize cooperation between various individuals and organizations, and 5) promote the development of human resources

As a shared value for the researchers who worked in the community space, we developed a model for the worker and the community, which was conceptualized as Coordination (Fig. 1) and Networking (Fig. 2).

### Participant selection

**Fig. 3:**
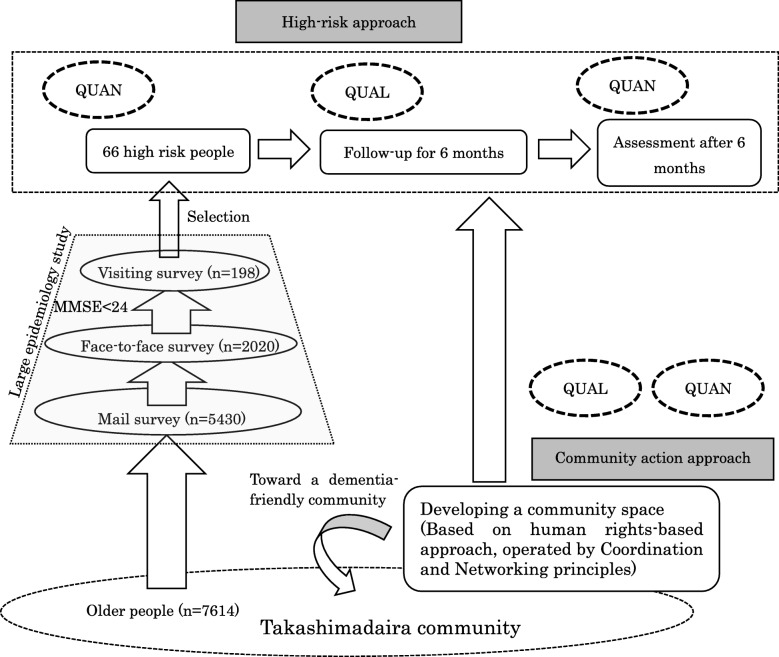
Overview of community-based participatory research in Takashimadaira, Tokyo

We selected 66 high-risk participants who were 70 years and older and living in the Tokyo metropolitan area. The flow of the study is described in Fig. 3.

Prior to this study, we conducted a three-step survey of all community residents who were 70 and over. As the 1st step, questionnaires were sent to a total of 7614 residents, and 5430 were retrieved. As the 2nd step, 2020 residents completed face-to-face surveys in the community center. This assessment included the Mini Mental State Examination (MMSE) [21, 22]. Those who scored below 24, which is a common cut-off criterion [23], became potential participants in the subsequent survey. As the 3rd step, a research team including a certified psychiatrist and a gerontologists or public health nurse visited the 198 participants in their homes.

A panel of specialists including a psychiatrist, gerontologists, and public health nurse then reviewed the health records for the 198 survey participants. Because of limited staff and time, we focused on high-risk participants. The criterion for judging an individual to be high-risk were 1) at least one social support need and 2) being at risk of not continuing to live in the community according to the psychiatrist, gerontologists, and public health nurse. This was judged according to a consensus process completed by an interdisciplinary board of experts, which consisted of experienced researchers.

### Setting

Our study was conducted in Takashimadaira, which is located in the northwest area of Metropolitan Tokyo. It is the largest housing complex district in Japan, and was built during the 70s, which was a high-growth period. An administrative corporation currently manages the housing complex. Because residents are not required to have a guarantor, which is Japanese business custom, many of the residents are older people who do not have relatives who they can rely on for financial support. The study period was from August 2017 to January 2018.

#### High-risk approach

The researchers, who were three public health nurses and six gerontologists, conducted follow-up assessments with the high-risk older participants. The researchers were encouraged to build partnerships with the participants. The researchers conducted counseling sessions with each participant more than once per month. The details regarding making contact and trust-building with the participants were determined by the individual researchers. We chose to allow the researchers to make these decisions independently for two reasons: 1) all of the researchers had more than 20 years of experience as public health nurses or gerontologists involved in the care of older people, and 2) because we thought that a structured or semi-structured approach for working with the residents of Takashimadaira might be regarded as cold, and we wanted to enable naturally occurring contact to facilitate the development of mutual trust.

#### Community action approach

We created a basecamp for our research that was also a comfortable place for community residents to spend time. Our community space was named COCOKARA Station (http://m.facebok.com/t.cocokarast/). The name comes from the Japanese words for heart (COCOro) and body (KARAda). It was open from 11:00 to 16:00 4 days a week and located in the center of the housing complex district. Anyone could visit the center and enjoy free tea, coffee, and a snack. Three to five staff, including at least one full-time researcher with a Ph.D., were present at COCOKARA Station during open hours.

We ensured that all of the staff were aware that human rights are universal such that all of the people in the world are entitled to them, and that these rights are inherent to the dignity of every human. The staff also learned about the PANEL framework. Our research team made a booklet, included in the supplementary materials, that explains the principles, philosophy, and operation of the experiment in Japanese, which is to be translated in English. For the community residents, we delivered a lecture about the human-rights-based approach and PANEL as part of a lecture series that we held every 2 months.

Prior to the opening of the center, we posted flyers to all of the residents of the housing complex district to explain that 1) this was research and not a commercial project, 2) no fee was needed to join the activities, and 3), the aim of the project was to construct dementia-friendly communities. An overview of the project was clearly displayed on the wall of the center. We also visited the community stakeholders before opening the center to explain the project and ask for their cooperation.

After the opening of the community space, we held meetings to collect feedback from the residents, i.e. what they liked and disliked about the services offered by the center. These meetings were 2–3 h long and took place every week.

### Data collection and data analysis

#### Quantitative assessment

##### Continuation of community living

The main outcome of this study was whether or not participants continued to live in the community after six months.

##### Dementia-related variables

***MMSE****.* As described before, we used the MMSE [21, 22] as the main measurement tool in the 2nd step of this study. Assessments were conducted by a psychologist or researcher who was supervised by a psychologist. The MMSE cutoff score was 23/24. This cutoff score is widely-used, including in the original work of Folstein as well as subsequent research [23].

*DSM-5 diagnosis of dementia*. In the survey interview (3rd step), a certified psychiatrist diagnosed participants according to the DSM-5 [24].

***CDR***. The Clinical Dementia Rating scale [25], which is widely used to measure the stages of dementia, was also scored by a certified psychiatrist.

***DASC-21***. The Dementia Assessment Sheet for Community-based Integrated Care System - 21 items (DASC-21) [26] was used to assess the progression of dementia symptoms. Because the DASC-21 is easy to score and allows observation-based ratings, it is widely used by caregivers who are part of the Japanese national dementia strategy. In this study, the researchers rated the participants before and after the observation period.

***NPI-Q***. Behavioral and Psychological Symptoms of Dementia (BPSD) were assessed during the initial and last phase of the intervention survey using the Neuropsychiatry Inventory-Questionnaire (NPI-Q) [27]. We used information obtained from family members, neighbors, and friends.

***J-ZBI_8***. In the case that family members were available to participate in the study, including both those who lived with participants and those who lived apart, care burden was also assessed using the short form of the Japanese version of the Zarit Burden Interview (J-ZBI_8) [28].

##### Mental-health related variables

***GDS-15****.* Depressive symptoms were also assessed using the 15-item Geriatric Depression Scale (GDS-15), which is a widely used screening instrument for depressive symptoms in older adults [29]. The score ranges from 0 to 15 and scores higher than five are considered to indicate the presence of depressive symptoms.

***WHO5-J-S****.* Mental well-being was assessed using the short version of the Japanese version of the WHO Five Well-Being Index (WHO-5) [30, 31].

##### Physical-health related variables

***Frailty****.* Frailty was assessed using the Kihon Checklist (KCL) [32], which was developed by the Japanese Ministry of Health, Labour, and Welfare to identify older people at risk of requiring care/support. The KCL is universally used by local governments to assess health and care needs in Japan. Satake et al. [33] found that total KCL scores were closely correlated with frailty, as defined in the Cardiovascular Health Study criteria. Total KCL cut-off scores of 7/8 and 3/4 were used to identify frailty and potential frailty, respectively.

***Access to a doctor****.* The participants were also asked whether they were registered with a home doctor, as this may be an indication of heightened access to medicine.

##### Community-related variables

***Interactions with neighbors****.* We asked participants about the frequency of their interactions with neighbors, and those who answered less than once per month were regarded as being in the low interaction group.

***Trust in neighbors****.* We also asked participants about the degree to which they trusted their neighbors, using the item“Do you trust your neighbors?” with a five-point Likert-type scale including “strongly agree”, “agree”, “neither”, “disagree”, and “strongly disagree” as responses. Those who answered “disagree” or “strongly disagree” were regarded as not trusting their neighbors.

##### Long-term care usage

We assessed whether participants were using a long-term care insurance service.

##### Need for social support

We evaluated the need for social support in terms of 9 domains: 1) Dementia disease diagnosis, 2) Medical check-ups for physical conditions, 3) Continuous medical care, 4) Daily living support, 5) Family support, 6) Housing support, 7) Long-term care insurance services, 8) Financial support, and 9) Rights protection.

##### Socioeconomic status

***Subjective SES***. We asked participants about their current socioeconomic status using a five-point Likert-type scale with “affluent”, “somewhat affluent”, “normal”, “somewhat poor”, and “poor” as responses. Those who answered “somewhat poor” and “poor” were regarded as having a financial disadvantage.

***Objective SES***. We also asked participants to report their income with six possible answers; “0”, “less than ¥1,000,000”, “less than ¥3,000,000”, “less than ¥7,000,000”, “less than ¥10,000,000”, and “over ¥10,000,000”. Those who answered“0” or “less than ¥1,000,000” were regarded as being low income.

##### Sociodemographic variables

We obtained basic sociodemographic information such as age, sex, years of education, marriage status, and co-habitation (whether or not the participant lived alone) via the mail-based questionnaires (1st step).

Concerning the amount of activity at the community space, we recorded the number of guests at the center each day and the number of medical consultations conducted at the center.

#### Qualitative assessment

Recording of interviews was not permitted in this study. The researchers were required to take field notes that were generally non-structured and that consisted of a description of the present situation and challenges faced by the participants, an assessment by the researchers, interventions, and episodes. The participants were continuously informed about the project with respect to the new community model and activities in the community space, and they were encouraged to visit the space. We aimed to assure the participants that the trained staff members who were part of the research team were always available to provide assistance and support.

#### Data analysis

For the quantitative data, we compared participants who continued to live in the community and participants who moved into institutions or hospitals. The quantitative characteristics of both groups were compared using a chi square test. We could not conduct multiple regression analysis because the sample size was not sufficiently large.

Qualitative data were analyzed via two processes: generalization of data obtained during follow-up assessments with each participant and generalization of data obtained from the minutes of the weekly meetings. Concerning the former process, the researcher first reviewed the field notes for each participant and made a summary of the follow-up period. Second, the researcher presented the summary in a 30-min presentation at a meeting and the entire research team discussed the function of the community space. Third, the researchers approved the merged functions during the meeting. Concerning the latter process, the research team reviewed the minutes of the weekly meetings to examine the function of the community space, and the merged functions were approved.

## Results

### Factors associated with high-risk participants continuing to live in the community

After the six-month follow-up period, 49 people remained in the community and 12 people had moved out of the community, i.e., into institutions (five people) or hospitals (seven people). We were not able to follow 5 participants for the entire follow-up duration. Table 2 compares the demographic variables of those who continued to live in the community with those who did not. Those who could not continue to live in the community had more unmet need in terms of social support, especially daily living support and housing support. In addition, their families perceived a heavier burden of care. Interestingly, a DSM-5 diagnosis of dementia, clinical dementia rating, physical health, mental health, and long-term care usage did not predict the outcome.

**Table 2:** The functions of the community space during the study period

Function	Examples
Activities involving other community workers	
Exchanging information	Every time our staff visited a participant, nobody answered the door and there was an unintelligible note on the door that appeared to have been written by the participant. Staff from existing community centers exchanged information regarding this issue in our space.
Coordination of community care	A participant was diagnosed with a life-threatening disease, but ran out of the hospital and refused to go back. Our staff arranged for the community physicians and staff from the existing community center to meet the participant in our space to make a care plan.
Activities involving other community workers and the individuals	
Anti-abuse action	A participant’s spouse seemed to be suffering from abuse, but was unable to talk about it at their home. Careful intake was conducted in our space, and we then reported the case to the local government.
Anti-stigma action	A participant was bullied because others regarded their forgetfulness as laziness. The researcher gave the participant a brief anti-stigma education.
Anti-poverty action	A participant was suffering from poverty and refused external help. Our staff collaborated with the community center to explain how to create a reliable safety net.
Outreach	When our staff visited a participant’s house, their caregiver was deeply confused about how to care for a person with frailty. The caregiver was referred to the community center for more information.
Activities involving just the individuals	
Education	A participant’s spouse was experiencing burnout because they were engaging in caregiving despite having been diagnosed with a serious illness. The researcher suggested that the couple access public help, educating them on the value of help-seeking.
Assistance with understanding the medical system	A participant’s family was suspicious because the general hospital decided to refer the participant to the neighboring outpatient clinic. The staff helped the family to understand the community medical system.
Emotional support for caregivers	A participant refused to go to the hospital and their family was exhausted from caregiving. The family talked about BPSD in our community space and received empathy from the staff.
Social participation	A participant who was homebound began to come to participate in activities.
Safeguarding a small amount of money	Although the participants enjoyed a community lunch club (organized by a different center), they often forgot to pay for it. Because the leader of the lunch club and the participants often meet at our space, we kept a small amount of money at our space to help the participants pay for lunch.
Day care	When a caregiver (who had denied that the participant they were caring for had dementia) suddenly died, the participant had no place to stay in the daytime and began wandering the community. Eventually, the participant stayed at our space during the day.

Concerning the 49 people who continued to live in the community, we assessed NPI-Q, DASC-21, and J-ZBI_8 scores before and after the follow-up period. Although NPI-Q (0.7 to 0.9, *p* = 0.228) and J-ZBI_8 (8.1 ± 7.7 to 9.0 ± 7.1, *p* = 0.531) scores were not significantly different, DASC-21 scores (34.6 ± 13.9 to 37.6 ± 15.0, *p* < 0.005) increased significantly, indicating that dementia had progressed.

### Development of a model of inclusive community space

The average number of daily guests to the center was 12.7. Medical consultations by a doctor were available on 17 days, and the average number of consultations per day was 3.4. A conference with the staff from the existing community centers was held once a month. A half-hour health lecture for the residents was delivered once per month.

Our community space served a number of different purposes for the residents, in addition to functioning as a basecamp for our observations. For example, when a participant’s only caregiver suddenly died, the participant had no place to stay in the daytime. The participant began wandering throughout the community because the caregiver had denied that the participant had dementia, resulting in a lack of social support. Thanks to a relative, the participant was diagnosed with dementia and began to receive appropriate social support. However, she was unable to understand her situation, and repeatedly came to our community space in the morning before it had opened. Although she was upset, it was possible to calm the participant down with conversation or an explanation provided by the researcher. Thus, the center functioned as a de-facto day care center.

Table 3 describes the functions of the community space, which included exchanging information, coordination of community care, anti-abuse, anti-stigma, and anti-poverty action, outreach, education, assistance understanding the medical system, emotional support for caregivers, social participation, safeguarding small amounts of money for residents, and day care. These functions were categorized into three categories: 1) activities involving other community workers, 2) activities involving other community workers and individuals, and 3) activities involving just the individuals. We described the various episodes that occurred at the centre carefully, omitting detail to ensure the privacy of the participants.

**Table 3:** Demographic variables of those who continued to live in the community compared with those who did not

		Continue to live in the community	Moved into institutions or hospitals		statistical value
number		49	12		
Sociodemographic variables					
Sex	Male	24 (49%)	4 (33%)		*p* = 0.343
	Female	25 (51%)	8 (67%)		
Age	65–74	81.9 ± 5.8	82.8 ± 4.7		*p* = 0.657
Education	≥ 9 years	13 (28%)	3 (27%)		*p* = 0.979
	< 9 years	34 (72%)	8 (73%)		
Living status	Living alone	21 (43%)	7 (58%)		*p* = 0.356
	Living with others	28 (57%)	5 (42%)		
Marital status	Married	29 (60%)	6 (55%)		*p* = 0.745
	Not married	19 (40%)	5 (45%)		
Dementia-related variables					
Dementia	DSM-5 dementia	12 (24%)	3 (25%)		*p* = 0.971
	not dementia	37 (76%)	9 (75%)		
CDR	0	5 (10%)	1 (8%)		
	0.5	19 (39%)	1 (8%)		
	1	22 (45%)	6 (50%)		
	2	2 (4%)	3 (25%)		
	3	1 (2%)	1 (8%)		
MMSE-J		20.2 ± 2.5	19.0 ± 3.0		*p* = 0.17
NPI-Q		0.9 ± 1.9	1.6 ± 2.2		*p* = 0.504
J-ZBI_8		7.9 + 7.5	19.7 + 3.8	*	*p* = 0.018
Mental-health related variables					
GDS	normal	17 (38%)	4 (33%)		*p* = 0.615
	mildly depressed	22 (45%)	5 (42%)		
	severely depressed	6 (12%)	3 (25%)		
WHO5-J-S		8.1 ± 4.0	6.2 ± 5.6		*p* = 0.183
Physical-health related variables					
Frailty	healthy	12 (26%)	2 (17%)		*p* = 0.846
	prefrailty	12 (26%)	4 (33%)		
	frailty	22 (48%)	6 (50%)		
Access to doctor	having GP	42 (88%)	10 (83%)		*p* = 0.655
	not having GP	6 (12%)	2 (17%)		
Community-related variables					
Communicating with neighbor	lower than 1/month	28 (64%)	8 (67%)		*p* = 0.846
	1/month and over	16 (36%)	4 (33%)		
Trust in neighbor	no trust	6 (13%)	4 (27%)		*p* = 0.088
	trust	40 (87%)	11 (73%)		
Socio-economic status					
Subjective disadvantage	present	19 (40%)	6 (50%)		*p* = 0.74
	absent	28 (60%)	6 (50%)		
Income	< 1,000,000 yen	9 (22%)	2 (20%)		*P* = 0.893
	over	32 (78%)	8 (80%)		
Long-term care usage					
using LTC		7 (14%)	4 (33%)		*p* = 0.124
not using LTC		42 (86%)	8 (67%)		
Need for social support					
Dementia disease diagnosis		31 (63%)	8 (67%)		*p* = 0.826
Medical check-up for physical conditions		8 (16%)	4 (33%)		*p* = 0.184
Continuous medical care		8 (16%)	4 (33%)		p = 0.184
Daily living supports		18 (37%)	9 (75%)	*	*p* = 0.017
Supports for their family		23 (47%)	8 (67%)		*p* = 0.221
Housing condition		2 (4%)	4 (33%)	**	*p* = 0.002
Lon-term care insurance		26 (53%)	7 (58%)		*p* = 0.743
Financial supports		7 (14%)	2 (17%)		*p* = 0.744
Rights protection		8 (16%)	4 (33%)		*p* = 0.184

* *p* < 0.05, **p < 0.01

## Discussion

Of the 66 high-risk individuals, 12 people were unable to continue living in the community during the observation period. Considering that previous studies have mainly focused on mortality or survival rates because of difficulties accessing detailed information, our study represents an important examination of the real-world environment experienced by older community residents.

Before conducting this study, we hypothesized that factors that might be associated with an inability to continue living in the community might include medical biomarkers such as 1) clinical dementia stage, 2) physical frailty, 3) poor mental health, and 4) not accessing long-term care. However, our data indicated that the significant determinants were not medical markers, but instead unmet needs regarding social support and care burden. The national dementia strategy of Japan consists of 7 pillars [5, 34]: 1) Raising awareness and promoting understanding of dementia, 2) Providing healthcare and long-term care services in a timely and appropriate manner as the stages of dementia progress, 3) Reinforcement of measures for younger onset dementia, 4) Support for those looking after people with dementia, 5) Creating age and dementia-friendly communities, 6) Promoting research and development and disseminating the results with regard to prevention, diagnosis, cures, rehabilitation models, and care models for dementia, and 7) Prioritizing the viewpoint of persons with dementia and their families. In hospitals, medical staff members sometimes focus mainly on healthcare and long-term care that concerns pillar 2. However, our results indicate that decreasing the family’s care burden (pillar 4), as well as the delivery of daily living support and housing support (pillar 5), is beneficial to community living. Thus, broadening the approach to care in medical settings may help older people to remain in the community for longer.

In this study, we used the CBPR approach to create a model of an inclusive community space with a human-rights-based approach, which is embodied in the PANEL framework. Our space acquired many functions on several levels. These included exchanging information, coordination of community care, anti-abuse, anti-stigma, and anti-poverty action, outreach, education, assistance understanding the medical system, emotional support for caregivers, social participation, safeguarding property, and day care. We grouped these functions into three categories: 1) activities involving other community workers, 2) activities involving other community workers and individuals, and 3) activity involving just individuals. These functions are consistent with the PANEL perspective. For any questions regarding legality, we consulted our legal counsel. For example, before we agreed to safeguard small amounts of money, we consulted our legal counsel and were advised that this activity was not legally problematic.

Because a guarantor, who is usually a family member or relative, is necessary to secure rental housing in Japan, socially isolated older people may find it difficult to rent an apartment. The housing complex in Takashimadaira is the largest housing complex owned by the Urban Renaissance Agency, which is an independent administrative agency that has built over 800,000 housing units since 1955, and does not require tenants to have a guarantor. As a result, many older isolated people live there. To examine the results of our present study, we proposed two context-mechanism-outcome configurations [35]. First, the members of the initial study population were aware that the district in which the housing complex was located was rapidly aging and had some anxiety about the future of the community (context). Because of this, the residents agreed to complete the public health survey, which was planned via a collaboration between the city officials and our institute (mechanism). As the result, we successfully conducted a very large-scaled epidemiology study (outcome). Accordingly, as the project progressed, the residents and researchers came to know one another (context). Based on this relationship, the building of the new community space was easily welcomed by the residents (mechanism). As the result, this CBPR project was realized (outcome). According to previous literature [36], such partnerships prioritize the generation of a shared vision together with shared values. In our study, based on the partnerships that were formed with the community residents, we were able to identify a function for enhancing age friendly communities in Japan.

However, several obstacles limit the generalization of our approach to different communities. First, research interventions might not be welcomed when residents do not have anxiety about the future of their community. Second, our project was large-scale and involved many researchers, and this might not be feasible in other communities.

During this study, researchers were strongly encouraged to build trust with people with dementia by visiting them at home. Information provided by the people with dementia was strongly respected, and was not considered to be inferior to information provided by family or other associated individuals. According to previous literature [37, 38], there is a discrepancy between caregiver and patient perspectives, and caregivers may have a more negative perspective regarding the function and daily life of patients. According to a qualitative analysis of in-depth interviews with people with dementia [39], a key factor in preserving personal dignity is engagement in meaningful activities within the safe and secure environment of the patient’s home. This previous report found that home is “a symbol of the identity the participant had built up over a lifetime”. One characteristic of our study is that assessments were mainly conducted at patient homes. Further research is needed to explore the factors that enhance the ability to continue living in the community.

### Strengths and limitations

One strength of this study is that while the participants were identified using the standard flow method of large-scale public health research, we observed the experiences of the participants from a research basecamp that was rooted in the community. However, our study had several limitations. First, the number of participants was 66, which is low when conducting statistical analysis. As a result, we could not conduct multiple logistic regression analysis. Second, the selection of the 66 high-risk individuals out of the 198 potential participants was not strictly operational; we relied on expert consensus rather than cutoff points for some psychometrics. Third, we did not employ brain imaging or blood tests to facilitate dementia diagnoses. Such tests could have enabled the visiting psychiatrist assessing neurological symptoms to rule out intracranial lesions or other physical conditions that lead to cognitive decline. Fourth, interactions with the researcher may have affected the main outcome, presumably by encouraging participants to continue community living. Fifth, we did not conduct a structured interview that was audio recorded or transcribed verbatim for our qualitative analysis.

## Conclusions

Merely providing healthcare and long-term care might not be sufficient to support community living in people with cognitive impairments. Daily living support and housing support should be provided in the context of a broad health services package. For this purpose, creating a comfortable community space for residents and community workers is essential.

## Supplementary information

**Additional file 1.** A booklet that explains the principles, philosophy, and operation of the experiment in Japanese.

## Data Availability

The datasets used and/or analyzed during the current study are available from the corresponding author on reasonable request.

## Abbreviations

BPSD: Behavioral and Psychological Symptoms of Dementia
CBPR: Community-Based Participatory Research
DASC-21: Dementia Assessment Sheet for Community-based Integrated Care System - 21 items
DFC: Dementia-Friendly Community
GDS-15: the 15-item Geriatric Depression Scale
J-ZBI: Zarit Burden Interview
KCL: Kihon Checklist
LTC: Lon Term Care
MMSE: Mini Mental State Examination
NPI-Q: Neuropsychiatry Inventory-Questionnaire
PANEL: Participation, Accountability, Non-discrimination, Empowerment, and Legality
SES: Socio Economic Status
WHO-5: WHO Five Well-Being Index
