# Supplementary material for: Everyday challenges facing high-risk older people living in the community: a community-based participatory study
Source: BMC Geriatr. 2020 Feb 17;20:68. doi: 10.1186/s12877-020-1470-y (PMC7027225; doi:10.1186/s12877-020-1470-y)
Supplement: Supplementary file 1 — Additional file 1. A booklet that explains the principles, philosophy, and operation of the experiment in Japanese. [file 12877_2020_1470_MOESM1_ESM.pdf]

認知症とともに暮らせる社会に向けて

コーディネーションとネットワーキングの手引き

地方独立行政法人 東京都健康長寿医療センター

東京都

目　次

1. はじめに …… 1
2. コーディネーション …… 2
（1）信頼関係の形成 …… 3
（2）総合的アセスメント …… 4
（3）情報共有 …… 5
（4）課題解決に向けた多職種協働 …… 5
（5）社会支援の調整 …… 6
3. ネットワーキング …… 18
（1）居場所としての機能 …… 18
（2）相談に応需できる機能 …… 20
（3）差別や偏見を解消し、住民同士の互助をつくりだす機能 …… 20
（4）連携を推進する機能（狭義のネットワーキング）…… 21
（5）人材を育成する機能 …… 23
4. おわりに …… 24
附録 地域での生活を支える社会資源 …… 26

認知症とともに暮らせる社会に向けて
コーディネーションとネットワーキングの手引き

発　行
東京都福祉保健局高齢社会対策部在宅支援課認知症支援担当
〒163-8001　新宿区西新宿2-8-1
電話　03-5320-4276
FAX　03-5388-1395

編　集
地方独立行政法人東京都健康長寿医療センター
東京都健康長寿医療センター研究所
自立促進と精神保健研究チーム　粟田主一
〒173-0015　板橋区栄町35-2
電話　03-3964-3241（代表）内線4222

平成30年（2018）3月　発行
禁無断転載
登録番号　(29) 483

1 はじめに

世界最高水準の長寿国であるわが国では、認知症とともに社会に暮らす人々の数も年々増加しています。それと同時に、大都市では、一人暮らしであるために、家族による支援が得られにくく、経済的にも困窮する高齢者が増えています。このことは、社会の中で孤立し、必要な情報やサービスにアクセスすることができず、経済的な困窮も相まって、地域生活の継続が困難になる認知症の高齢者が増加することを意味しています。そのようなことから、東京都では、平成28年度～29年度に、「認知症とともに暮らせる社会」のモデルをつくりだすことを目的に、「認知症とともに暮らせる社会に向けた地域ケアモデル事業」を実施しました。

この事業は、観察研究「生活実態調査」と介入研究「コーディネーションとネットワーキング」で構成される研究事業です。この研究の結果、①認知機能の低下が、生活機能の低下とともに、身体的・精神的な健康状態の悪化、社会的孤立、経済的困窮と深く関連していること、しかも、そのような身体的・精神的・社会的リスクの複合化が、認知症の初期段階に生じていること、②大都市の大規模住宅地には、単独世帯をはじめ、家族による生活支援が得られにくい高齢者が数多く暮らしていること、同時に、認知機能の低下が、社会的交流の減少と関連し、それが情報や社会支援サービスへのアクセスをさらに困難にさせている可能性があること、③実際に、認知症とともに地域に暮らす高齢者の多くは、医学的な診断や介護保険サービスの利用にはつながっておらず、数多くの複合的な社会支援ニーズが存在するにも関わらず、必要な支援にアクセスできていないこと（おそらく、その多くは、情報の欠如と社会支援の利用を援助する担い手の欠如によること）が明らかになりました。また、④多職種協働によるコーディネーションが社会支援の利用を促進すること、⑤5つの機能を担う地域拠点の活動が、人々の認知症についての理解を深め、認知症とともに生きる人々の権利についての意識を高め、「認知症とともに暮らせる社会」の創出に向けた活動を促進することが明らかになりました。

ここで言うコーディネーションとは「本人の視点に立った、社会支援の統合的調整」を意味しており、ネットワーキングとは「社会支援を相互に提供することを可能にする地域づくり」を意味しています。本書の目的は、これらの研究成果を踏まえて、コーディネーションとネットワーキングの方法を解説することにあります。

「認知症とともに暮らせる社会」とは、認知症の有無に関わらず、障害の有無に関わらず、地域に暮らすすべての人々が、地域の大切な一員であると認識され、未来に向けて、希望と尊厳をもって暮らすことができる社会にほかなりません。本書が、大都市のさまざまな地域で展開される「認知症とともに暮らせる社会」の実現に寄与することができれば幸いです。

東京都健康長寿医療センター研究所
研究部長　**粟田 主一**

2 コーディネーション

認知症や障害などによって生活のしづらさに直面しているときに、本人の視点に立って、生活の継続に必要な社会支援を統合的に調整することをコーディネーションと呼びます。また、コーディネーションの役割を担う人をコーディネーターと呼びます。

現在ある制度の中でも、たとえば地域包括支援センターや居宅介護支援事業所の業務の中心はコーディネーションにほかなりません。また、認知症地域支援推進員、認知症支援コーディネーター、介護支援専門員はコーディネーターであり、認知症サポート医もコーディネート医としての役割が期待されています。そして、認知症初期集中支援チームは、多職種協働によるコーディネーションを実践する事業です。

しかし、ここでは、現存する制度や職種に関わらず、より一般的な意味でコーディネーションという言葉を用い、コーディネーションの業務を担う人をコーディネーターと呼ぶことにします。

ところで、認知症や障害の有無に関わらず、住む家があること、食べる物があること、着る服があること、必要な情報を得ること、医療や介護などのサービスを利用できること、趣味やレジャーを楽しむこと、自分の暮らし方を自分で選択できること、可能な限り健康を保つことは、人が人として、尊厳をもって、あたりまえに暮らすための諸条件です。人が人として、尊厳をもって、あたりまえに暮らすための諸条件のことを「権利」と呼びます。そのような意味で、コーディネーターは、「権利の行使を促進する」伴走者（パートナー）としての役割を果たします。

ですから、認知症の本人や家族を支援するコーディネーターは、「認知症」と「権利」について深い認識をもつ必要があります。コーディネーターの役割を担う人は、認知症支援の実践経験をもつ保健・福祉の専門職に限定すべきかどうかについては議論があります。たとえば、個人情報を守るという観点からは、職業上守秘義務が課せられている専門職であることが望ましいかもしれません。また、責任の明確化という観点からは、公的事業としてコーディネーションを行うべきかと思われます。しかし、実際には、民間事業や市民活動の中で、職種に限らず、その人の経験や学びを通して、コーディネーションが実践されているという現実もあります。

そこで、ここでは、「認知症」と「権利」にフォーカスをあてた研修を受け、日々の実践の中で、「認知症」について学び、認知症とともに生きる人々の「権利」について意識を高めることに努める人であることをコーディネーターの基本要件にしたいと思います。

通常、コーディネーションのプロセスには、(1) 信頼関係の形成、(2) 総合的アセメント、(3) 情報共有、(4) 課題解決に向けた多職種協働、(5) 社会支援サービスの調整という５つの柱があります（**図１**）。これらの活動の時間的流れは複合的であり、しばしば同時進行的でありますが、この５つの柱が基軸となってコーディネーションが展開され、社会支援の統合的調整という目標が達せられます。

図１　コーディネーションのプロセス

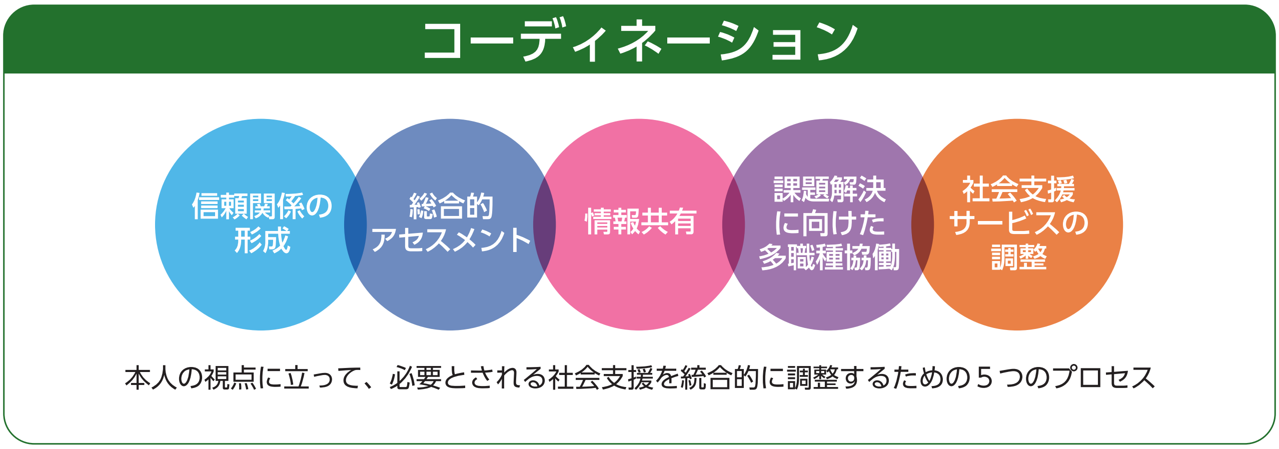

ここで言う「本人の視点に立って」という言葉は、パーソン・センタードという考え方に一致します。英国のウースター大学のドーン・ブルッカー教授は、パーソン・センタードが意味するポイントをVIPS（Value/Individuality/Perspective/Social Environment）という4つのポイントで明確化させています（**表1**）。

表1　VIPS

**V**	年齢や認知能力にかかわらず、全ての人の存在自体に絶対的な価値があることを認めること
**I**	個人の独自性を尊重してアプローチすること
**P**	認知症をもつ人の視点から世界を理解すること
**S**	心理的ニーズを満たし、相互に支え合う社会的環境を提供すること

「VIPSですすめるパーソンセンタードケア」、ドーン・ブルッカー著、水野裕監修（クリエイツかもがわ）より

以下に、コーディネーションのプロセス（5つの柱）について解説します。

（1）信頼関係の形成

コーディネーションの第一歩は信頼関係を形成すること、すなわち、“信頼できるパートナーシップを築くこと”にあります。なぜならば、「本人の視点に立って、社会支援の調整を行う」には、本人の思い、希望、心配ごと、困りごと、悩みごと、これまでの人生の歴史、体験してきたことなどを知り、理解することからはじめなければなりませんが、それができるのは、語り合う2人の間に信頼関係が形成されているからでしょう。

しかし、信頼関係は、通常は短い時間で形成されるものではなく、日々の語り合いや、体験の共有など、“生き生きとした人と人との関わり合い”の積み重ねの中で形成されるものです。そのような時間の流れの中で、コーディネーターは、“本人の視点に立って考える”“本人の立場に身を置いて考える”という姿勢を保ち続ける必要があります。

何をもって信頼関係が形成されているかを判断することは簡単ではありませんが、**表2**に掲げられているような本人の体験が、信頼を意味するものなのかもしれません。決して急ぐことなく、根気よく、“信頼できるパートナーシップを築く”ための時間をつくりだしていきましょう。

表2　信頼という体験とは何か

— 私の思いを語ることができる
— 私の話に耳を傾けてくれる
— 私の困りごとの相談にのってくれる
— 私の不安や心配を理解してくれる
— 私の立場に立って考えてくれる
— 私がわからないことを一緒に考えてくれる
— 私の人生の歴史を理解してくれている
— 私の希望を語ることができる
— 私の希望を理解してくれている

（2）総合的アセスメント

コーディネーションを行うには、本人の思いや希望を理解するとともに、現在の生活状況全般において何が課題であるかを客観的に把握する必要があります。そのようなプロセスを総合的アセスメントと呼びます。

認知症の総合的アセスメントを行う場合には、認知症の一般的特性に関する知識が不可欠です。すなわち、認知症は、何らかの脳の病的変化によって、認知機能が障害され、それによって生活に支障が現れた状態ですが、それと同時に、さまざまな身体的・精神的・社会的な生活課題に直面し、それによって生活の継続そのものが困難に陥るリスクが高まった状態であることを理解する必要があります（**図2**）。

したがって、現時点で、どのような認知機能に障害が認められるか、生活面ではどのようなことに支障があるのか、身体的健康状態はどうか、精神的健康状態はどうか、社会的にはどのような状況におかれているのかということを把握する必要があります。また、質の高い適切なサービスを確保するためにも、これらの背景にはどのような認知症疾患があるのかを理解することも重要です。

図2　認知症の全体像―総合的アセスメントを行うために

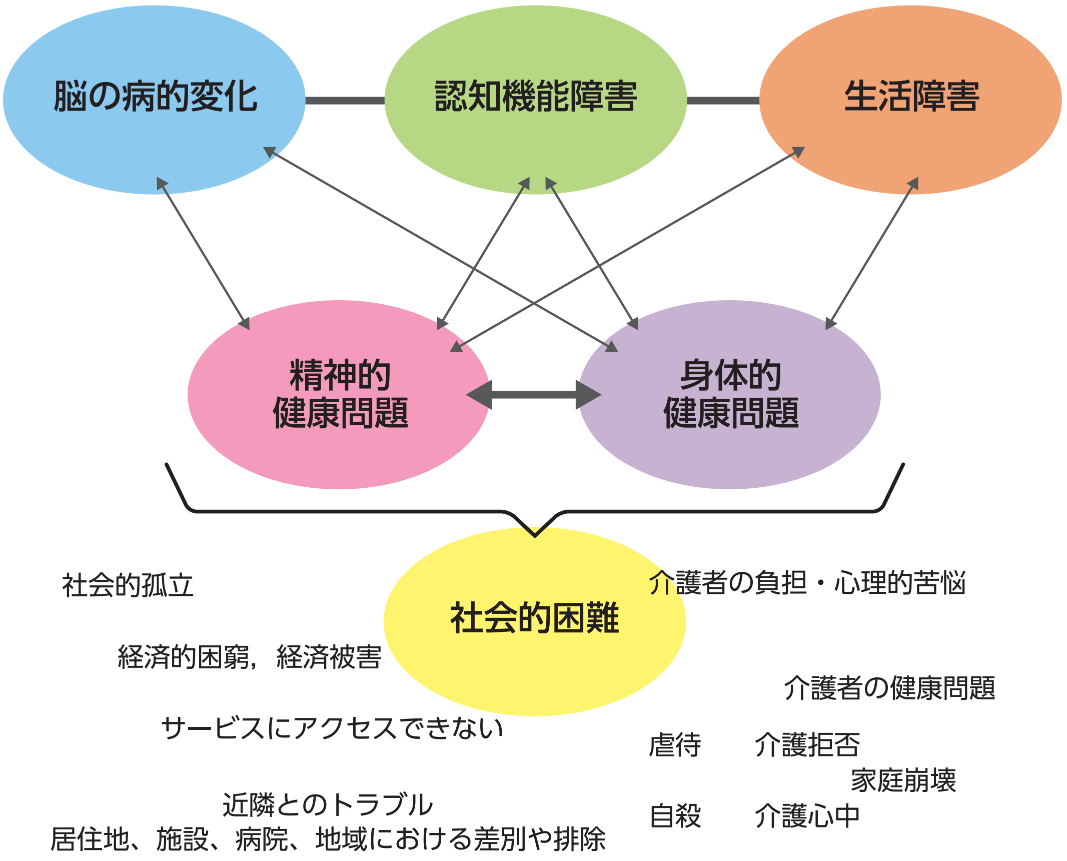

(3) 情報共有

本人の視点に立って、生活の継続に必要な支援のあり方を考えるためには、第一に、本人や家族と情報を共有する必要があり、第二に多職種間で情報を共有する必要があります。

認知症の本人や家族に情報を提供するにあたっては、よく配慮された、質の高い情報を、わかりやすく、正確に伝える必要があります。たとえば、ワーキングメモリや近時記憶などの認知機能に低下が認められる場合には、複雑な情報や長い話は負担が大きく、情報共有が困難になりがちです。そのような場合には、認知機能の低下によく配慮して、大切な情報を選択し、短い言葉で、わかりやすく伝えることが大切です。パンフレットを利用したり、メモを書いたりするなどの工夫も必要でしょう。

また、本人や家族の不安が強い場合には、安心感を与えられるような配慮も必要です。ただ単に、認知症であることを伝えたり、診断名を伝えたり、障害の性質を伝えることよりも、本人が心配していることや、希望していることに対して、役に立つ情報を提供することからはじめることが大切な場合もあります。

保健、医療、介護、生活支援、家族支援、居住支援、経済支援、権利擁護の支援など、複合的な支援が必要とされる場合には、多職種で情報を共有し、支援のニーズと課題解決の方法を検討していく必要があります。

(4) 課題解決に向けた多職種協働

課題解決に向けた多職種協働とは、多職種協働チームが、本人の視点に立って、本人の主体性を尊重しながら、可能性のある課題解決に向けて、本人とともに歩んで行こうとするアプローチです。このようなアプローチによって、重層的な生活課題に直面し、不安を感じている人も、寄る辺なさを感じている人も、抑うつ状態にある人も、絶望感に圧倒されている人も、自己効力感や生きる意欲を回復し、その人なりの課題解決に向けて、希望と尊厳をもって、地域社会の中で共に生きることが可能になります。尚、ここでは、支援を必要としている対象が誰であるのか、認知症である本人なのか、介護する家族なのか、そのことを明確にしてディスカッションすることが重要です。

課題解決に向けた検討を行うには、概ね以下の手順で多職種協働会議を運営します。

①会議の設営

会議設営の担当者が時間と場所を定め、メンバーを招集し、資料等を準備します。

②司会進行と記録

司会進行役と記録係を定めます。司会進行役は、メンバー全員が自由かつ公平に発言できるように配慮します。記録係は、会議の内容を記録し、次回会議で資料として活用できるように管理します。

③支援対象者と担当者(コーディネーター)の確認

司会進行役は、支援対象者が誰であるか、支援対象者の担当者(コーディネーター)が誰であるかを確認します。

④情報共有

コーディネーターは支援対象者の状況(総合的アセスメントの結果など)を説明し、多職種のメンバー間で情報を共有し、整理します。

⑤課題の明確化

情報共有のプロセスを通して、課題（支援ニーズ）を明確化させます。

⑥解決目標の設定

明確化された課題から、いくつかの課題を選択し、具体的な解決目標を定めます。

⑦解決策の案出

定められた具体的な解決目標に向けて、できるだけ多くの解決策を案出します（ブレインストーミング）。

⑧支援方針の決定

案出された解決策の中から、現実に「実行できそうな方法」「うまくいきそうな方法」を選択して支援方針を決定し、支援計画を立案します。

⑨支援計画の策定と実施

支援方針や支援計画について、コーディネーターは本人や家族の意向を確認しながら、支援計画をさらに具体化させて、支援を実施します。

⑩モニタリング

コーディネーターは支援の実施状況や計画について記録し、次回会議で報告し、さらなる支援の必要性等について検討します。

(5) 社会支援の調整

多職種でのディスカッションを通して、多様な課題と社会支援ニーズが明確化されます。一般的に、抽出される頻度が高い社会支援ニーズは以下の９つの領域に分類することができます。

①認知症等の医学的診断

認知症が疑われる場合には、それが「認知症の状態か否か？」、「背景にある要因は何か？」という２段階の医学的な診断が必要となります（**図3**）。そのような診断が、その後の質の高い支援を確保する上で重要な意味をもっています。認知症等の診断が必要とされる場合、通常はかかりつけ医と連携して診断へのアクセスを確保します。かかりつけ医がいない場合には、認知症サポート医や認知症疾患医療センターなどの医療機関への受診を勧奨します。いずれの場合も、コーディネーターは、認知症について、診断の必要性について、受診可能な医療機関について、本人や家族にわかりやすく情報を提供します。

ご本人が診断を受けたくないと希望される場合には、まずは優先される社会支援ニーズを定め、それに対するサービスの調整からはじめます。また、受診に際して家族の協力が得られにくい場合には、受診同行に関する生活支援（後述）の調整が必要な場合もあります。

図３　認知症の医学的診断のプロセス

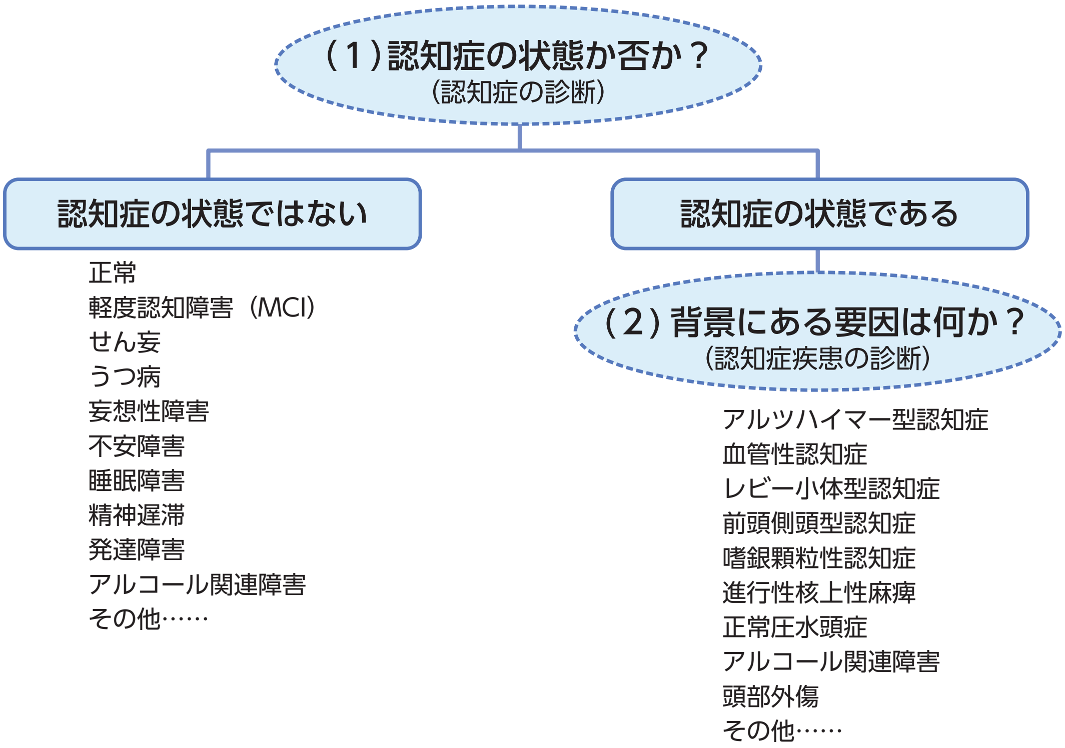

②身体的健康状態の医学的評価

認知症では、高齢である場合が多いこと、背景にある脳の病的変化（認知症疾患）が運動麻痺やパーキンソニズムなどの身体症状をもたらす場合があること、服薬管理や栄養管理などの支障が身体的健康状態を悪化させている場合があることなどから、身体的健康状態の医学的評価が必要な場合が少なくありません。

たとえば、かかりつけ医がいない場合には、身体的健康状態を評価してもらう機会そのものがまったくない場合があります。かかりつけ医がいても、受診そのものが途絶えていたり、服薬管理の障害のために持病である高血圧症や糖尿病が悪化していたり、買い物や食事の準備の障害、口腔機能の低下、経済的困窮などを背景に低栄養や貧血が認められることも少なくありません（**表３**）。

身体的健康状態を医学的に評価する必要がある場合には、通常は、かかりつけ医と情報を共有したり、認知症サポート医、認知症疾患医療センター等への受診を勧奨したりすることによって医療サービスへのアクセスを確保します。ご本人が医療機関を受診したくないと希望される場合には、往診や訪問看護を利用して、身体的健康状態を評価する方法もあります。受診に際して家族の協力が得られにくい場合には、受診同行の生活支援についても検討します。

表３　認知症の高齢者によく見られる身体症状、身体疾患

A. 身体症状
①神 経 症 状：パーキンソニズム、不随意運動、パラトニア、痙攣、運動麻痺
②廃用症候群：筋委縮、拘縮、心拍出量低下、低血圧、肺活量減少、尿失禁、便秘、誤嚥性肺炎、褥瘡
③老年症候群：転倒、骨折、脱水、浮腫、食欲不振、体重減少、肥満、嚥下困難、低栄養、貧血、ADL低下、難聴、視力低下、関節痛
④そ　の　他：不整脈、睡眠時呼吸障害、排尿障害、嗅覚障害、慢性硬膜下血腫、悪性症候群
**B. 身体疾患**
①全身疾患：脱水症、低栄養、電解質異常など
②呼吸器疾患：誤嚥性肺炎、慢性閉塞性肺疾患、肺結核、肺癌など
③循環器疾患：高血圧症、うっ血性心不全、虚血性心疾患、心房細動など
④消化器疾患：消化性潰瘍、腸閉塞、肝硬変、アルコール性肝障害、癌など
⑤腎疾患：腎硬化症、高血圧症性腎症、糖尿病性腎症、慢性腎不全など
⑥内分泌・代謝疾患：糖尿病、甲状腺機能低下症など
⑦泌尿器科疾患：下部尿路障害、尿路感染症、前立腺肥大症・癌など
⑧整形外科疾患：骨粗鬆症、骨折など
⑨皮膚科疾患：褥瘡、白癬、疥癬など
⑩眼科疾患：視力障害、白内障、緑内障など
⑪耳鼻咽喉科疾患：難聴、めまいなど
⑫神経・筋疾患：脳血管障害、パーキンソン症候群など
⑬口腔疾患：う蝕、歯周病など

③継続医療の確保

医学的な評価によって認知症疾患や精神疾患や身体疾患が認められ、かつ継続医療が必要と判断されるがそれが確保されていない場合には、通常、かかりつけ医と連携して必要な継続医療を確保します。また、かかりつけ医がいない場合には、認知症サポート医や認知症疾患医療センター等と連携して、継続医療が確保されるように調整します。ご本人が医療機関を受診したくないと希望される場合には、往診や訪問看護を利用して、継続医療を確保する方法もあります。受診に際して家族の協力が得られにくい場合には、受診同行の生活支援についても検討します。

④生活支援

日常生活を営むために必要とされる多様な支援は、認知症とともに生きる本人が、その人らしく、その人の人生を主体的に生きていく上で重要な意味をもっています。そのような支援には、介護保険給付サービスではカバーされにくいものが多いことから、「介護」とは区別して、「生活支援」という用語が用いられています。通常は、このような生活支援は家族がその担い手になっていますが、家族よる生活支援が得られにくい状況では（例：単身世帯など）、家族に代わる生活支援の担い手の確保が求められます。ここには、以下のような支援が含まれます（**表４**）。

表4　生活支援の内容

a. 人と人とのつながりをつくる支援
b. 社会とのつながりをつくる支援
c. 医療機関における受療に関する支援
d. 家事行為に関する支援
e. 金銭管理や服薬管理など私的領域に関する日常生活支援
f. その他の支援

a. 人と人とのつながりをつくる支援

高齢者の生活支援において、「見守り」という言葉がよく使われます。これは、定期的に本人の自宅を訪問したり、電話をかけたりすることなどによって、本人の安否を確認したり、必要なときに対応できる体制を整えておくことを意味しています。しかし、それは単に見守ることではなく、このような支援によって、本人の思い、心配、不安、希望、夢などに耳を傾け、語り合う機会がつくられ、信頼関係（パートナーシップ）が醸成され、情緒的サポート（例：困っているときに相談にのる、具合が悪いときに相談にのるなど）を確保する基盤がつくられるものでなければなりません。そのような意味で、コーディネーションの最初のプロセスである「信頼関係の形成」と密接に関連しています。それ故に、ここでは、「見守り」というよりも、「人と人とのつながりをつくる支援」と呼ぶことにします。

b. 社会とのつながりをつくる支援

社会とのつながり（社会参加）は、その人なりの人生を、希望と尊厳をもって暮らしていくための基盤です。しかし、認知機能障害や生活障害は、社会とのつながりを希薄にし、社会的孤立を促進する重大な要因になっています。認知症とともに生きる人が、人々と出会える場につながれるようにすることはコーディネーターの重要な役割です。そのような場には、サロン、認知症カフェ、趣味やレジャー、文化や芸術、仕事やボランティアなどがあります。その中でも、認知症とともに生きる本人同士が出会うことには、とても重要な意味があります。わが国では、認知症とともに生きる本人同士が主体的に出会い、自由に語り合い、それを政策や地域づくりに反映させ、地域社会の創出に参加することをめざす「本人ミーティング」が、各地で少しずつ立ちあがり始めています。後述する地域拠点で、「本人ミーティング」を開催することもできるでしょう。

c. 医療機関における受療に関する支援

自分の体調が悪いときに看病してもらったり、医療機関に同行してもらったり、受診の手続きをしてもらったり、医師の説明を一緒に聞いたり、入院の際の手伝いをしてもらったり、安否を確認してもらったりする支援は、本人が求める生活支援の中でも頻度が高い支援であることが明らかにされています。要介護認定を受けている場合には訪問介護などのサービスでカバーできる場合もありますが、そうでない場合には家族に代わる同行支援の担い手を個別に検討する必要があります。

d. 家事行為に関する支援

掃除、洗濯、草取り、電球の交換、買い物、食事の準備など家事行為に関する支援は、要介護認定を受けている場合には、訪問介護などの居宅サービスでカバーすることができますが、未認定である場合や、サービスを十分な頻度で利用できない場合には、介護保険外の生活支援を個別に検討する必要があります。介護保険外の自治体事業として実施されている配食サービスや民間サービス、地域の中で展開されている食事会、NPO法人やボランティア団体の生活支援サービスを活用することができるかを検討します。

e. 金銭管理や服薬管理など私的領域に関する日常生活支援

認知症では、軽度の段階で、手段的日常生活動作（IADL）の障害として金銭管理や服薬管理の障害が認められることが少なくありません。自分で銀行に行って預貯金の出し入れをしたり、1カ月分の生活費を管理したり、家賃や光熱費の支払いをしたり、確定申告等の税金関係の手続きをしたり、サービス利用のための契約手続きをしたり、決まった時間に決まった分量の薬を服用したり、こうした生活行為を行うのにしばしば支援が必要になります。通常は、家族がこれを支援していますが、単身世帯では、それを支援する担い手が確保できず、そのために日常生活に支障が現れたり、トラブルが生じたり、健康状態を悪化させたりすることがあります。しかし、このような生活行為は、認知機能が低下している高齢者であっても、援助を希求しにくい、あるいは他人の手を借りたくないという“私的領域”に属するものかと思われます。

一般的には、後述する日常生活自立支援事業（地域福祉権利擁護事業）の利用を検討することになりますが、しかし、こうした制度的支援のみではきめ細かな日常生活支援は困難かと思われます。後述するように、このような制度的サービスは、日常生活支援を可能にする地域づくり（ネットワーキング）を基礎にして、その利用を調整する必要があります。

f. その他の支援

上記以外にも多様な生活支援のニーズが考えられます。たとえば、これまで自分で自動車を運転してスポーツクラブに通ったり、釣りに出かけたりすることが生きがいであった人が、運転免許を返納することになったためにそれができなくなった場合、その人の生きる希望をどのように支援することができるのか。その答えは簡単には出せません。しかし、後述する地域拠点では、希望と尊厳をもって暮らすための新たな生活支援のあり方を創出する機能を発揮することも期待されます。生活支援のあり方は、認知症の有無に関わらず、障害の有無に関わらず、希望と尊厳をもって生きる権利をいかにすれば行使できるのか、という観点から考えていく必要があります。

⑤家族支援

家族は、通常、認知症とともに生きる本人の生活支援の大切な担い手です。しかし、生活支援の領域は広範であり、それらを担う介護者の負担は並大抵のものではありません。介護者自身も高齢であったり、病気や障害がある場合には、その負担はさらに大きくなり、精神的に苦悩したり、健康を害したり、介護者自身が社会的に孤立し、経済的に困窮し、困難な状況に陥ってしまうことも少なくありません。コーディネーションでは、家族介護者の状況もアセスメントし、家族介護者自身も人生の主人公として生きることができるように、家族に対する社会支援の調整を行う必要があります。

家族に対する社会支援の基本は、評価的サポート（ねぎらうこと）、情緒的サポート（困りごとや心配事の相談にのること）、情報的サポート（必要な情報を提供すること）、手段的サポート（実際にサービスの利用を調整したり、介護サービスを利用したり、経済支援に係る諸制度を利用したりすること）と整理することができます。この中で、情緒的サポートと情報的サポートは家族支援の根幹です。

情緒的・情報的サポートは、一般的には、専門職による支援（個別相談、心理教育、家族教室など）、家族同士の相互支援（家族会など）、専門職・家族・市民を含む相互支援（ケアラーズカフェなど）によって提供されます。コーディネーターは、自らが情緒的・情報的サポートの提供者であると同時に、家族がこれらの社会支援につながることができるように調整する役割を果たします。後述する地域拠点において、ケアラーズカフェを立ち上げることもできるでしょう。

介護者が仕事をしている場合、しばしば介護のために仕事の継続が困難となり、離職を考えることがあります。介護離職は介護者自身の将来にわたる経済状況にも重大な影響を及ぼします。仕事をしている介護者には、「職場と家庭の両立支援」に関する情報（**表5**）を早めに提供することも重要です。

表5　職場と家庭の両立支援に関する情報

育児・介護休業法（育児休業、介護休業等育児又は家族介護を行う労働者の福祉に関する法律）では、以下のことが定められています。

1. 介護休業、介護休暇
要介護状態にある家族を介護するために、事業主に介護休業、介護休暇を申し出ることができます。事業主はその申し出を拒むことができません。

2. 所定外労働、時間外労働、深夜業の制限
要介護状態にある家族を介護するために、所定外労働、時間外労働、深夜業の制限について請求があった場合、事業主は上記の労働をさせてはなりません。

3. 事業主が構ずべき措置
事業主は介護休業中の待遇などを定め周知させる処置を講ずるように努めなければなりません。また、職業家庭両立推進者を定めなければなりません。

4. 国等による援助
国等は、事業主の相談及び助言、給付金の支給などを行うことができます。

厚生労働省ホームページ（http://www.mhlw.go.jp/stf/seisakunitsuite/bunya/0000130583.html）参照

⑥居住支援

住まいは地域生活を継続する基盤です。しかし、認知機能障害や生活障害によって、住み慣れた「住まい」を失う状況に直面する場合は決して少なくありません。生活を継続するための「住まい」を安定的に確保するための支援を「居住支援」と呼びます。ここには、「現在の居住環境を整備する支援」と「新たに住まいを確保する支援」が含まれます。

a. 現在の居住環境を整備する支援

現在の居住環境（建物の構造、段差、温度、日当たり、水回り、音、調理器具、整理整頓の状況）をアセスメントし、必要な助言と利用できる社会支援サービスについて情報を提供します。整理整頓や清潔保持の困難（例：室内の混乱、ゴミ屋敷など）や近隣とのトラブルは、生活を継続できる「住まい」の喪失につながる可能性を高めます。そのような場合には、本人の思いを聴き、関係者と協働して、多職種協働チームで課題解決に向けた検討を行う必要があります。

b. 新たに住まいを確保するための支援

居宅系サービスを利用しても在宅生活の継続が困難な状況にあるときは、本人や家族とともに、生活支援のある住まいの確保を支援します。ここには、サービス付き高齢者向け住宅、有料老人ホーム、認知症対応型共同生活介護（グループホーム）、介護老人保健施設、介護老人福祉施設（特別養護老人ホーム）、介護療養型医療施設、軽費老人ホーム（ケアハウス）などがあります。しかし、その人がおかれている状況（例：経済状況、家族や身寄りがいない、医療ニーズが高いなど）によって、利用できるサービスが限定される場合があります。特に経済状況（**表6**）は重要であり、状況によっては居住支援・生活支援を提供するNPO法人等との連携が必要な場合もあります。

表6　入所入居施設一覧

種類	内容	要件	期間	費用	備考
特別養護老人ホーム	入浴、排せつ、食事等の介護その他の日常生活上の世話、機能訓練、健康管理及び療養上の世話を行う	要介護3以上	長期	約10万円～18万円	医療依存度が高いと対応不可
介護老人保健施設	看護、医学的管理の下における介護及び機能訓練その他必要な医療並びに日常生活上の世話を行う	要介護1以上	概ね3ヶ月	約8万円～15万円	入所中に他医療機関の受診は不可。薬価に制限あり。
介護療養型老人保健施設	看護、医学的管理の下における介護及び機能訓練その他必要な医療並びに日常生活上の世話を行う	要介護1以上	長期	約10万円～16万円	医療の必要性の高い利用者を受け入れることを要件
介護療養型医療施設<br>※平成30年から介護医療院が創設されます	慢性疾患を有し、長期の療養が必要なものに対して、必要な医療サービス、日常生活における介護、リハビリテーションなどを行う	要介護1以上	長期	約10万円～20万円	重度の方が優先。2020年までに廃止される方針。
認知症対応型共同生活介護	認知症であるものに、その共同生活を営むべき住居において、入浴、排せつ、食事等の介護その他の日常生活上の世話及び機能訓練を行う	要支援2以上	長期	約16万円～20万円	認知症の診断が必要
軽費老人ホーム(A型)	無料又は低額な料金で、老人を入所させ、食事の提供その他日常生活上必要な便宜を供与する	60歳以上	長期	収入に応じ1万円～10万円	自炊が出来ない高齢者向け。要介護状態になったら退所。
軽費老人ホーム(B型)	無料又は低額な料金で、老人を入所させ、食事の提供その他日常生活上必要な便宜を供与する	60歳以上身寄りなし	長期	収入に応じ1万円～10万円	自炊が出来る程度の健康状態、要介護状態になったら退所
ケアハウス	自立した生活を継続できるよう構造・設備等の面で工夫されており、各種相談、食事サービスの提供、入浴サービスの提供	身の回りのことは自立	長期	収入に応じ6万円～17万円	介護型ケアハウスは要介護1以上
介護型有料老人ホーム	老人を入居させ、入浴、排せつ若しくは食事の介護、食事の提供又はその他の日常生活上必要な便宜であって厚生労働省令で定めるものの供与をする事業を行う	各施設による	長期	約15万円～35万円	プランにより初期費用がかかる
住宅型有料老人ホーム		各施設による	長期	約15万円～35万円	介護サービス利用により費用が高額になる場合がある
健康型有料老人ホーム		自立～軽介護	長期	約15万円～35万円	プランにより初期費用がかかる

※サービス付高齢者向け住宅は、賃貸住宅であり、介護度の低い比較的元気な高齢者のための施設。安否確認と生活相談の2つのサービスを受けられる。したがって、生活支援や介護サービスを受けたい場合には、賃貸契約とは別にサービスの利用契約を自分で行う必要がある。

畠山 啓「4.高齢者が利用できる社会保障制度、社会資源」精神科治療学第32巻増刊号(2017)を改変

⑦介護保険サービスの利用支援

介護保険サービスの利用が課題解決に有効である考えられる場合には、本人・家族に介護保険制度の情報をわかりやすく説明した上で、地域包括支援センターと連携して、要介護認定申請等のサービス利用に向けた支援を行います（**表7**、**図4**）。また、実際にサービスを利用する場合に、介護支援専門員や介護サービス事業所のスタッフとも協働して、本人が希望と尊厳をもって暮らすためのトータルな支援のあり方を検討します。

表7　介護保険で利用できる居宅系サービス

サービス利用の形態	サービスの種類
家庭で受けるサービス	訪問介護、訪問入浴介護、夜間対応型訪問介護、定期巡回・随時対応型訪問介護看護、訪問看護、訪問リハビリテーション、居宅療養管理指導
通所等により受けるサービス	通所介護・地域密着型通所介護（デイサービス）、認知症対応型通所介護（認知症デイサービス）、通所リハビリテーション（デイケア）、小規模多機能型居宅介護、看護小規模多機能型居宅介護、短期入所生活介護（福祉系ショートステイ）、短期入所療養介護（医療系ショートステイ）
その他のサービス	福祉用具貸与、福祉用具購入費の支給、住宅改修費の支給

図４　介護サービスの利用に係る流れ

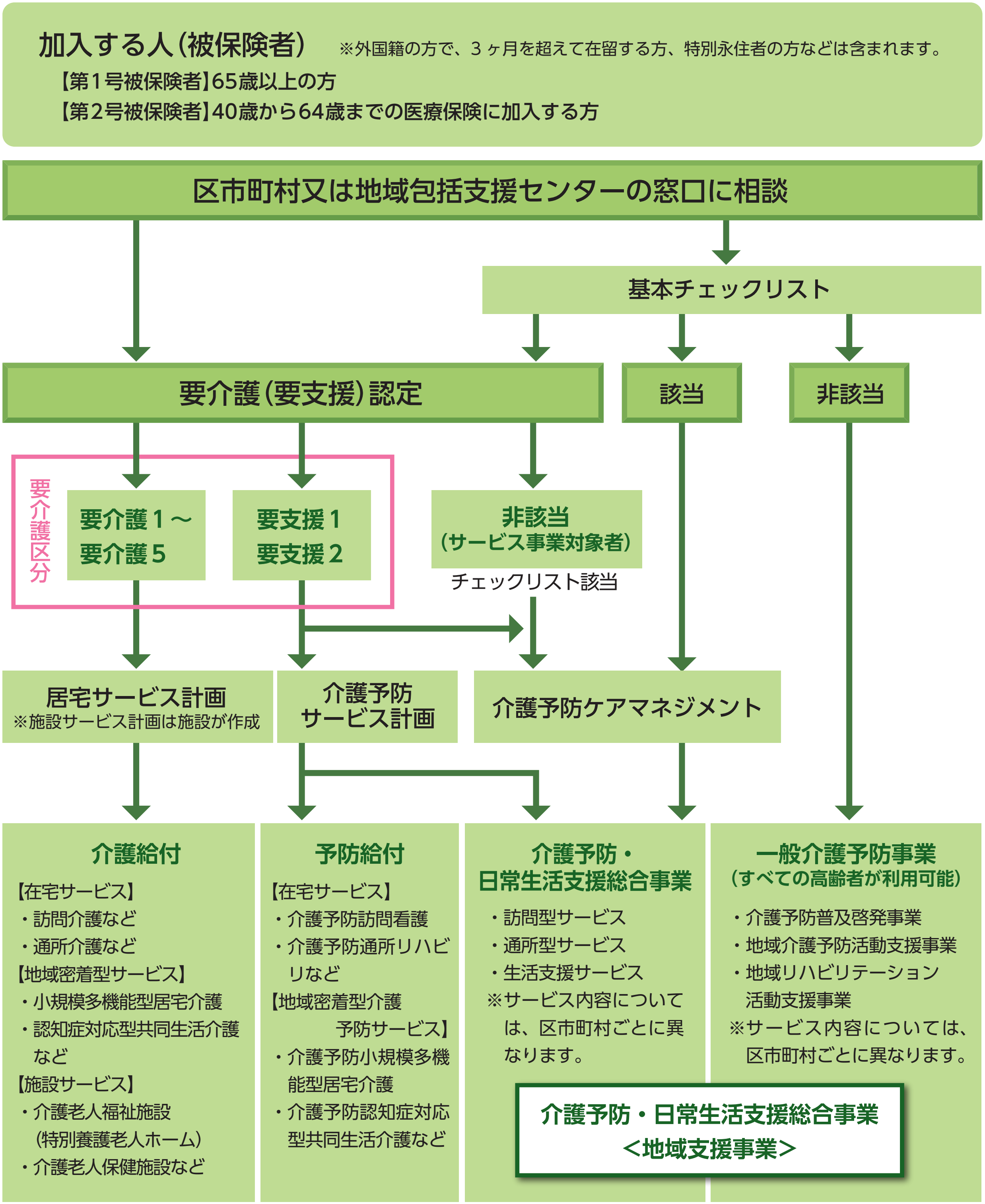

※「介護保険制度」（東京都福祉保健局高齢社会対策部介護保険課）を参考に作成
※上記の図は一般的な介護保険の利用の手順をお示ししたものです。詳細は、お住まいの区市町村へお問い合わせください。

⑧経済的な支援

経済的な理由から本来必要なサービスの利用が困難な状況にある場合には、経済支援のための諸制度に関する情報を提供し、サービスの利用を調整する必要があります。医療サービスや介護サービスの利用料を減額する制度としては、高額療養費制度や高額介護サービス費制度があります。また、疾病や障害の状況によっては、自立支援医療制度、難病医療費助成制度、特別障害者手当、精神障害者保健福祉手帳、身体障害者手帳、障害年金などの制度を利用できる場合があります。さらに、経済的な理由から、日常生活の継続そのものが困難な場合には、生活保護制度の利用について検討することができます。高額療養費制度の申請は加入している医療保険によって異なるため、加入している医療保険の窓口に相談します。高額介護サービス費、自立支援医療制度、難病医療費助成制度、特別障害者手当、精神障害者保健福祉手帳、身体障害者手帳、障害年金制度、生活保護制度は区市町村の担当窓口に申請します。なお、後期高齢者医療制度における入院費用の概算額の目安を**表8**に示します。

表8　入院費用の概算額

	項目	生活保護	低所得Ⅰ	低所得Ⅱ	1･2割負担	3割負担
保険内	医療費自己負担額	0	15,000	24,600	44,400	100,000
	食費	0	9,000	18,900	32,400	32,400
保険外	病衣・タオル等	36,000	36,000	36,000	36,000	36,000
	オムツ代	39,000	39,000	39,000	39,000	39,000
	小　計	75,000	99,000	118,500	151,800	207,400
合　計	差額室料なし	75,000	99,000	118,500	151,800	207,400
	差額室料1,000円の場合	105,000	129,000	148,500	181,800	237,400
	差額室料5,000円の場合	225,000	249,000	268,500	301,800	357,400
	差額室料10,000円の場合	375,000	399,000	418,500	451,800	507,400

※後期高齢者医療制度による負担分類
※医療費3割負担分は、80,100+（総医療費-26,700円×1%）を概ね100,000円と算出
※病衣タオル代は、日額1,200円、オムツ代は、1,300円で算出
畠山 啓「4.高齢者が利用できる社会保障制度、社会資源」精神科治療学第32巻増刊号（2017）より引用

⑨権利擁護に関する支援

権利擁護に関する支援は生活支援の一部とみなすこともできます。財産の管理、サービス利用の契約手続き、日常の金銭管理などを自分一人の判断で行うことが困難であり、かつ生活支援の担い手となる家族が不在の場合には、日常生活自立支援事業（地域福祉権利擁護事業）や成年後見制度の利用を検討することができます（**表9**）。また、経済被害の可能性がある場合には消費者被害センターや警察・弁護士等と連携し、虐待の可能性がある場合には、地域包括支援センターおよび区市町村の担当課と連携して対応を検討します。

ただし、これらの公的制度は、先に述べたような日常生活支援そのものをカバーできるものではありません。日常の生活支援を可能にする地域づくり（ネットワーキング）を基礎にして、制度の利用を調整することが求められます。

表9　日常生活自立支援事業と成年後見制度

制度の名称	内容
日常生活<br>自立支援事業	認知症、知的障害、精神障害等のために判断能力が十分ではなく、自分ひとりで福祉サービスの利用契約が困難な場合、本人との契約に基づいて、福祉サービスの利用援助、日常的な金銭管理、書類等の預かりサービスなどの支援を行う制度。本事業を利用するには、「本人が本事業の内容を理解し、契約を締結する能力があること」が条件となっている。
成年後見制度	認知症、知的障害、精神障害等により、判断能力が十分でない人に対して、本人の権利を守る援助者を選任し、財産管理や身上監護（身の回りの世話）に関する契約等の法律行為全般を支援する制度。法定後見制度と任意後見制度がある。法定後見制度は、家庭裁判所によって選任された成年後見人等（成年後見人、保佐人、補助人）が、本人の利益を考えながら、本人に代わって契約などの法律行為をしたり（代理権）、本人が自分で法律行為を行うときに同意を与えたり（同意権）、本人が成年後見人等の同意を得ないで行った不利益な法律行為を後から取り消したり（取消権）することによって、本人を保護・支援する制度。任意後見制度は、本人に十分な判断能力があるうちに、将来判断能力が不十分な状態になった場合に備えて、予め自ら選んだ代理人（任意後見人）に、自分の財産管理・身上監護に係る法律行為の代理権を与える契約（任意後見契約）を公証人の作成する公正証書によって締結しておく制度。

認知症疾患診療ガイドライン2017（医学書院）p186-187参照

3 ネットワーキング

社会支援を相互に提供していくことを可能にする地域社会の構造を創り出していくことをネットワーキングと呼びます。「地域づくり」、「まちづくり」とほぼ同じ意味です。ここでは、特に、「認知症の有無に関わらず、障害の有無に関わらず、希望と尊厳をもって暮らすことができる社会」をつくるという共通の目標に向けて、認知症の本人、家族、地域に暮らす人々、地域を構成する多様な組織・団体が、連携をつくりだし、共に活動することのすべてをネットワーキングと呼ぶことにします。

ネットワーキングを効果的に実践するには、以下の５つの機能を発揮する地域の拠点が必要でしょう。

①居場所としての機能

認知症の有無に関わらず、障害の有無に関わらず、「みんなが居心地よく、だれでも自由に」過ごすことができる。

②相談に応需できる機能

認知症を含め、多様な生活課題をもって暮らす人々に対して、専門的な観点からも相談に応需することが可能であり、かつ必要に応じて、適切な社会資源（保健・医療・福祉・介護に係る諸機関等）につなぐことができる。

③差別や偏見を解消し、住民同士の互助をつくりだす機能

認知症に関する正しい知識の普及とともに、多様な活動（イベントなど）を通して差別や偏見が解消され、社会支援を相互に提供することができる人と人とのつながりを創り出すことができる。

④連携を推進する機能（狭義のネットワーキング）

社会支援を提供する多様な組織・団体の相互連携（顔の見える連携）を推進することができる。

⑤人材を育成する機能

「認知症」と「権利」について深い認識をもった支援の担い手を育成することができる。

ここでは、上記に示すような５つの機能を担う場所を「地域拠点」と呼び、その具体的な活動の例を示しながら解説します。

（1）居場所としての機能

- 「認知症とともに暮らせる社会の創出」を開設理念とし、認知症の有無に関わらず、障害の有無に関わらず、「みんなが居心地よく、だれでも自由に」過ごせる居場所であることを、リーフレット、パンフレット、ポスター、ホームページなどを用いて、地域の人々に周知します（**図5**、**図6**）。
- 開設準備の段階から、地域の多様な組織・団体（地域包括支援センター、医療機関、介護保険事業所、民生委員、町内会、コミュニティカフェや認知症カフェなどを運営する多様な組織・団体）に、上記の開設趣旨について周知をはかり、協力を求めます。
- 地域拠点は可能な限り常設とし、ローテーションを組んで運営スタッフを配置し、地域に住む人々が自由に時間を過ごせる開室日を可能な限り多く確保します。
- 地域拠点を運営するスタッフは、「認知症」と「権利」にフォーカスをあてた研修を受け、日々の活動においても、認知症の有無に関わらず、障害の有無に関わらず、「みんなが居心地よく、だれでも自由に」過ごせる居場所をつくりだせるように配慮します（**図7**）。

図5　地域の拠点を周知するためのリーフレット

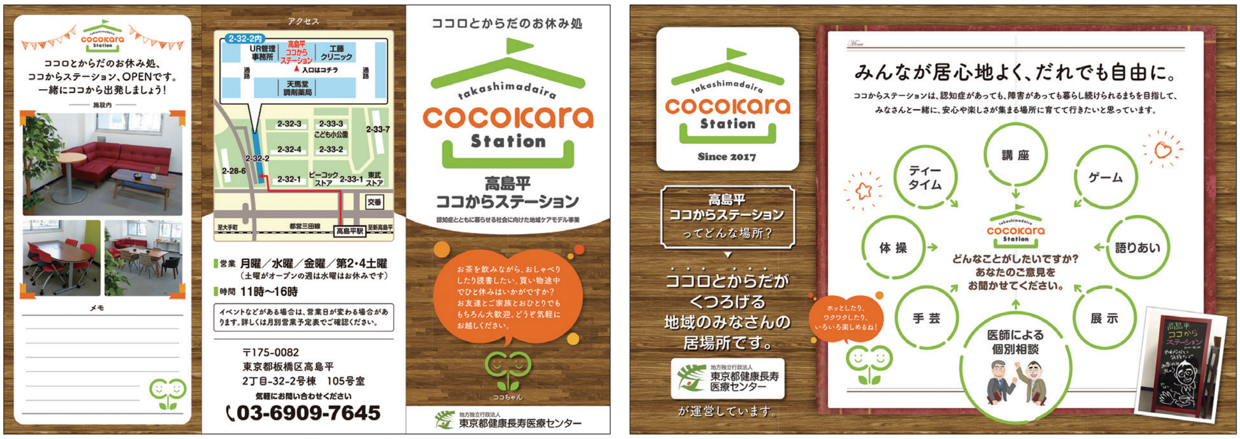

図6　高島平ココからステーション

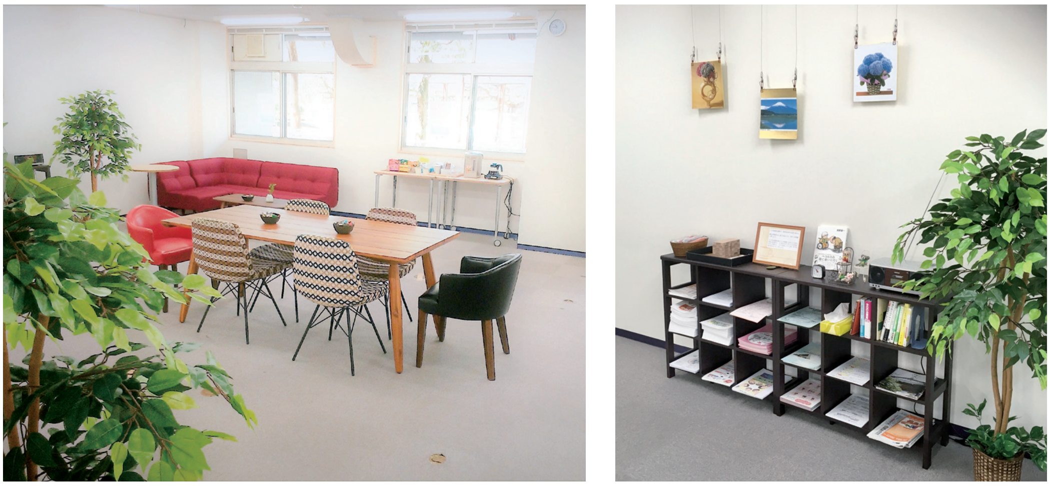

図7　高島平ココからステーション利用時のルール

ココは自由な場所で、特別な決まりはありません。

誰もが気持ちよく過ごせるように、他の人を尊重しましょう。

ココでは切実な困りごとが話題になることもあるかもしれません。人に知られたくないこともあるかもしれません。ココでお話したことは、外では話さないようにしましょう。

(2) 相談に応需できる機能（図8）

- 認知症を含め、多様な生活課題をもって暮らす人々に対して、専門的な観点からも相談に応需することができ、かつ必要に応じて、適切な社会資源（保健・医療・福祉・介護に係る諸機関等）につなぐことができるように、保健・医療・福祉等の専門職を運営スタッフに含めます。
- 特に、長年にわたって第一線で活躍され、定年退職を迎えられた専門職がスタッフに加わることで、質の高い相談機能を発揮することができます。
- 週の中に特定の日を設けて、医師による相談が受けられるようにするなどの工夫も有用です（例：ドクターマンデー）。実際、こうした日を設けることによって、ここに数多くの相談者が来場するようになります。
- 相談に応需する人には、認知症についての深い知識をもち、認知症とともに生きる人々の権利について高い意識をもつこと、地域の社会資源や制度等に精通していることが求められます。そのようなことが担保されていれば、地域に暮らす人々のさまざまな生活課題の相談に応じることができます。
- 相談を契機に、必要な社会資源と連携することができるように、日ごろから「顔の見える連携づくり」を進めておくことが重要です。

図8　高島平ココからステーションの相談スペース

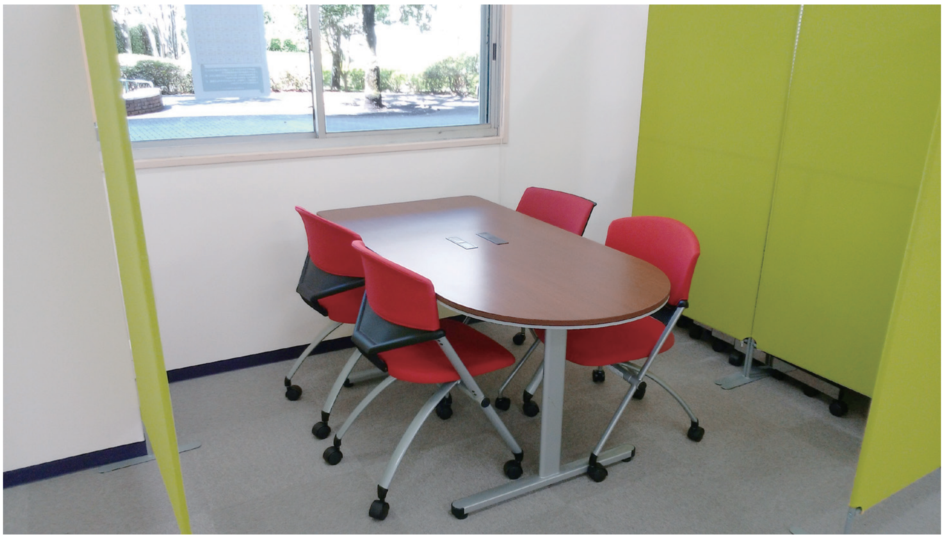

(3) 差別や偏見を解消し、住民同士の互助をつくりだす機能

- 「認知症についての正しい知識」の普及とともに、多様な活動（イベントなど）を通して、社会支援を相互に提供することができる人と人とのつながりを創り出すことが重要です。
- たとえば、地域住民を対象に、「認知症についての知識」、「認知症とともに生きるということ」、「人権を護ること」にフォーカスをあてた講座を開催することによって、認知症とともに生きる人に対する配慮が醸成されます。また、認知症だけではなく、さまざまな人が興味をもつ、さまざまなテーマの講座やイベントを開催することによって、多様な人々が気軽に立ち寄り、認知症の本人や家族と交流し、来場者同士のつながりが自然につくりだされます（例：ココからミニ講座、**図9**）。

- ときには、来場者の間に認知症に対する偏見や差別が見られることもありますが、そのようなときには運営スタッフの配慮によって、認知症である本人との交流の中で、次第に偏見や差別が解消され、来場者同士の間でも、「みんなで居心地よく、だれでも自由に」過ごせるようにするための配慮が芽生えはじめます。
- 「認知症とともに暮らせる社会の創出」という理念を見失うことなく運営を続けることによって、地域拠点が差別や偏見を解消し、互助を創出する場に成長していきます。

図9　ココからミニ講座とさまざまなイベント

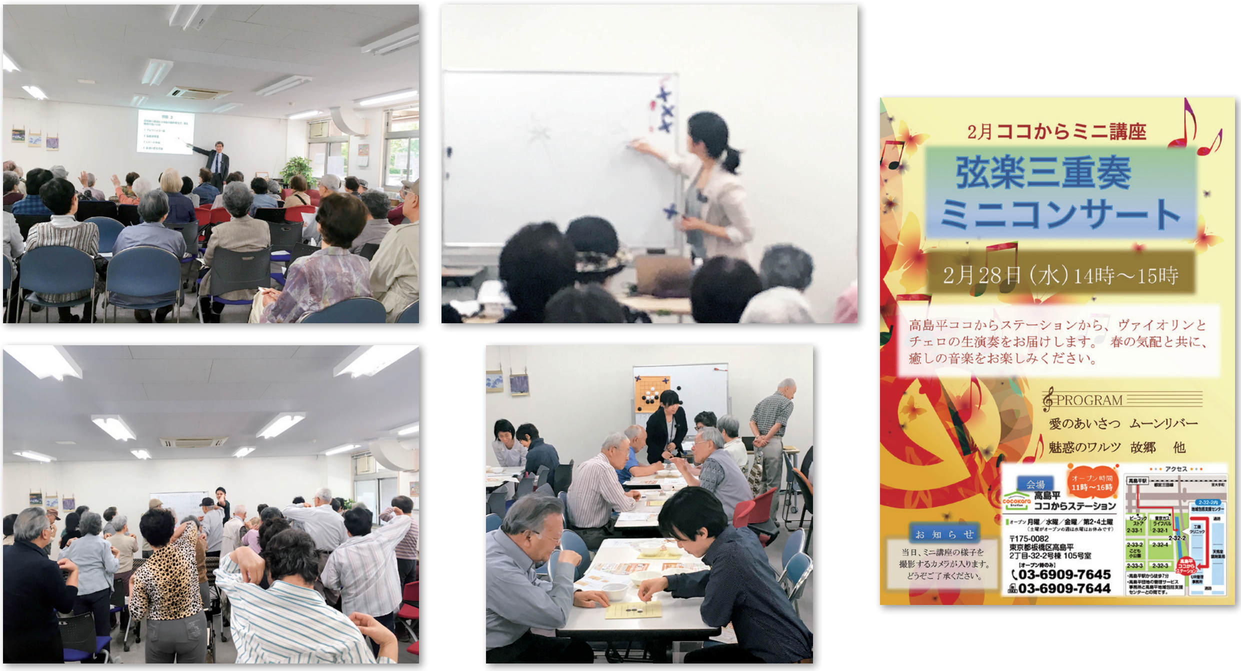

（4）連携を推進する機能（狭義のネットワーキング）

- 「認知症とともに暮らせる社会の創出」という理念のもとで、地域社会のさまざまな組織・団体と情報を共有したり、イベントを開催したり、地域の多様なネットワーク会議に参加したり、そのような会議を新たに立ち上げたりすることによって、社会支援を提供する多様な組織・団体との相互連携が推進されます。
- 例えば、住宅供給を目的とする企業のスタッフが、地域拠点で開催されるミニ講座、イベント、研修会への参加や、認知症の本人との対話が契機になって、「認知症にやさしい」住宅づくりへの取り組みがはじまります。
- 地域ですでに進められている居場所づくりの取り組み（例：板橋コミュニティスペース連絡会、**図10**）との連携によって、地域の多様な認知症カフェやサロン活動との横のつながりができ、活動全体が活性化されます（**図11**）。
- 生活支援を提供する既存のNPO法人等との連携も進み、生活支援の担い手となるボランティア育成や認知症サポーターの活用に関する取り組みを協働で実施できるようになります。

● 地域包括支援センター、居宅介護支援事業所、訪問看護ステーション、介護サービス事業所、訪問診療所等と協働しながら、個別支援を統合的に展開することが可能になります。

図10　板橋コミュニティスペース連絡会が作成したパンフレット

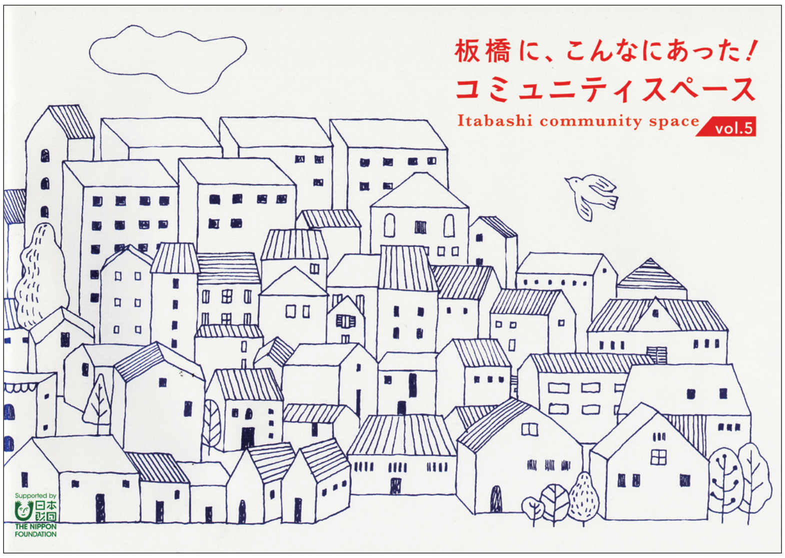

図11　他の団体や市民活動の情報を共有できるようにするための掲示スペース

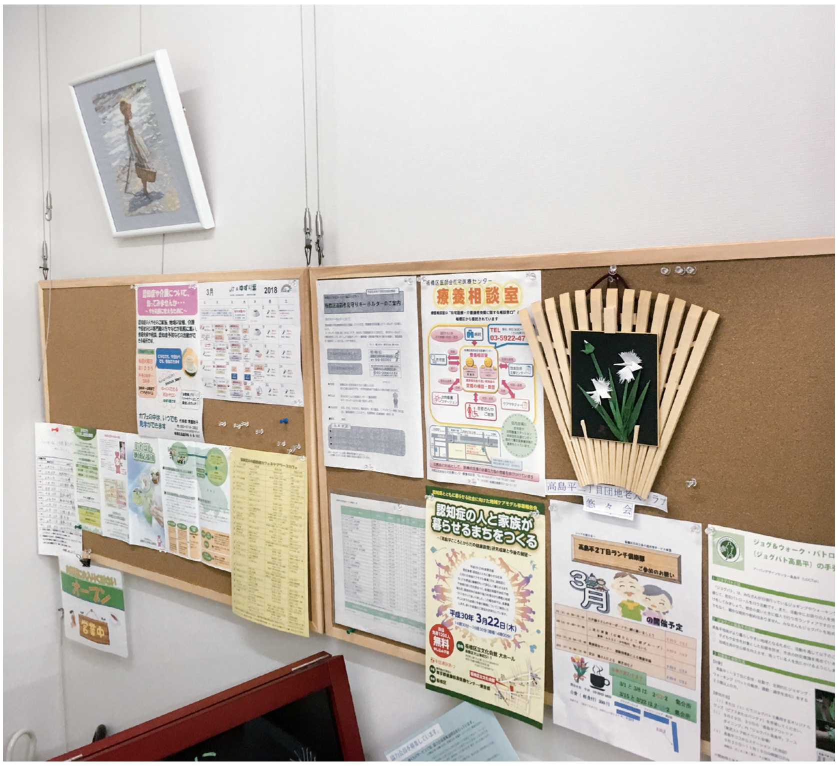

(5) 人材を育成する機能

- 「認知症」と「権利」にフォーカスをあてた研修会を定例的に開催することによって、受講者の認知症について理解を深め、認知症とともに生きる人々の権利についての意識を高めることができます (**図12**)。
- 研修会では、さまざまな領域の専門職、認知症である本人、認知症の人を介護する家族、地域で多様な活動をしている人々に講師をお願いすることによって、多様な観点から、「認知症」と「権利」にフォーカスをあてた意見交換をすることができます。
- 「認知症」と「権利」について高い意識をもつ支援の担い手を育成することは、「認知症とともに暮らせる社会」をつくる根幹になります。

図12 「認知症」と「権利」にフォーカスをあてたコーディネーター&サポートワーカー研修会

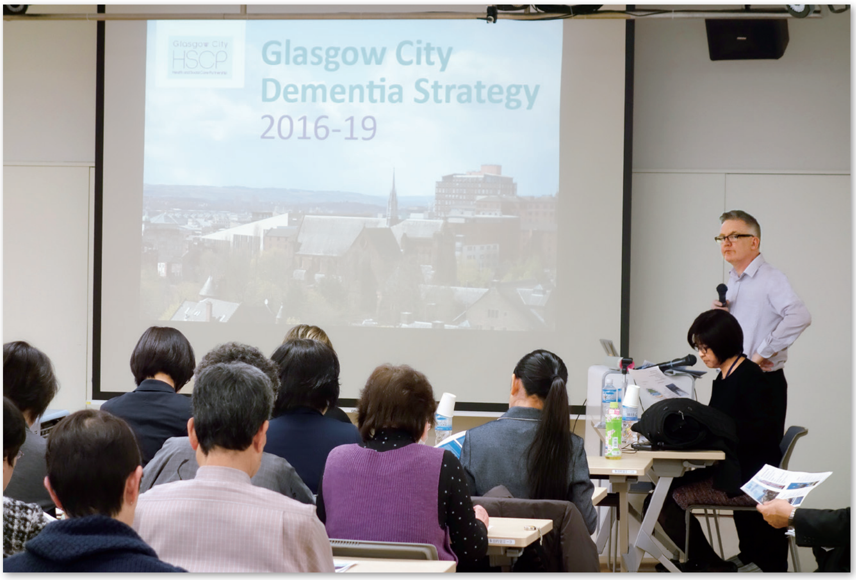

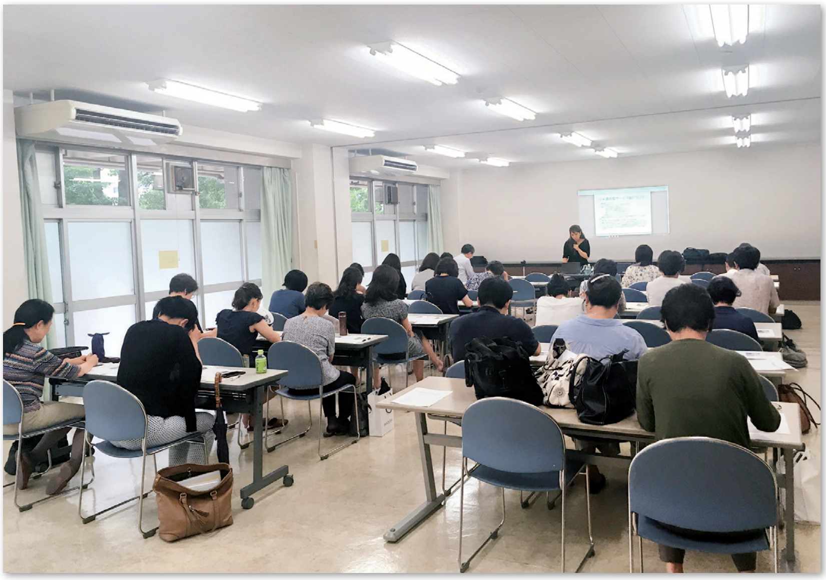

4 おわりに

コーディネーションとネットワーキングは車の両輪であり、認知症とともに暮らせる社会を創出するために必要な、最低限の基本モデルかと思われます。

しかし、このような活動を通して改めて実感されることは、社会支援のネットワーク、特に、一人暮らしの人や社会的に孤立している人をサポートする居住支援と生活支援のネットワーク、家族介護者をサポートする家族支援のネットワークが決定的に不足しているということです。地域拠点は、先に述べた5つの機能を通して、「認知症とともに暮らせる社会」に必要な新たな社会資源をつくりだし、ネットワークの継続と発展に努めていかなければならないでしょう。

また、コーディネーションとネットワーキングは、権利ベースのアプローチ（Rights Based Approach, RBA）という観点から考案した「認知症とともに暮らせる社会」を創出するための方法論と考えることもできます。RBAの基本的な考え方は、権利が実現されない状況にフォーカスをあてて、その要因を徹底的に分析し、権利保有者（社会支援を必要とする人）が権利を行使できるように、責務履行者（社会支援を提供する役割を担う人）がその責務を果たせるように、状況を変化させることにあります。

RBAを認知症施策に一早く取り入れたスコットランドでは、2009年に国連障害者権利条約に準拠した「認知症とともに生きる人々とケアラーのための権利章典」を作成し、PANELと呼ばれる原則を示しました。2015年に世界保健機関はこれを世界に紹介し、認知症施策に取り入れることを推奨しています。本書の最後に、参考資料としてPANEL原則の全訳を掲載しておきます（**表10**）。

表10　PANEL原則（参考資料）

P：Participation（参加）

1. 認知症の本人とケアラーは、情報にアクセスし、彼らに影響を及ぼす決定に参加する権利が行使できるように、必要な支援を提供される権利を有する。
2. 認知症の本人とケアラーは、コミュニティーにおける娯楽、レジャー、文化的生活にアクセスすることによって、可能な限り自立した生活を営む権利を有する。
3. 認知症の本人とケアラーは、事前意思決定を含め、ケアニーズのアセスメント、ケア・支援・治療の計画・決定・調整に参加する権利を有する。
4. 認知症の本人とケアラーは、ウエルビーングと権利の行使に影響を及ぼす政策の策定・実施に参加できるように支援される権利を有する。

A：Accountability（責任）

5. 認知症の本人とケアラーは、日常生活のすべての側面で、場所を問わず、個人の尊厳、信念、環境、プライバシーへの完全な尊重を含め、人権と基本的自由を享有する。
6. 認知症の本人のケアと治療に関与する公的・私的組織、ボランタリー組織、個人は、本人の人権を尊重し、保護し、充足する責任をもち、これが担保されるように適切なステップを踏まなければならない。

N：Non-discrimination and Equality（非差別、平等）

7. 認知症の本人とケアラーは、年齢、障害、性、人種、性的志向、宗教的信念、社会的地位など、いかなる理由によっても差別されない権利を有する。

E：Empowerment（エンパワメント）

8. 認知症の本人とケアラーは、保護、リハビリテーション、励ましを提供する適切なレベルのケアにアクセスする権利を有する。
9. 認知症の本人は、生活のすべての側面において、最大限の自立、身体的・精神的・社会的・職業的能力、十分な包摂と参加を確保・維持できるよう援助される権利を有する。
10. 認知症の本人とケアラーは、コミュニティーにおける教育と生涯学習の機会にアクセスできる権利を有する。
11. 認知症の本人は、自律性、保護、ケアを促進するための社会的・法的サービスにアクセスできる権利を有する。
12. 認知症の本人は、最も質の高いサービスを確保するために、認知症と人権について適切なトレーニングを受けた専門職とスタッフによって提供される保健・福祉サービスを利用する権利を有する。

L：Legality（法の遵守）

13. 認知症の本人とケアラーは、完全な範囲で人権を尊重され、保護され、充足される権利を有する。1998年の人権法に明示されていることに加えて、以下のことが含まれる。
    - ・尊厳と安全が保障された生活を営み、搾取、暴力、身体的・精神的・性的虐待を受けない権利。
    - ・社会的保護を含む適切な標準的生活を含む、経済的、社会的、文化的権利。
    - ・最大限確保できる標準的な身体的・精神的健康を得る権利。
14. 認知症の本人とケアラーは情報を得て、意思決定に参加する権利を有する。権利の行使が認められない場合、効果的な訴えと明確な手続きを通して法的救済を追及する権利を有する。
15. 認知症の本人は、診断が何であれ、他のすべての人と同様の市民的・法的権利を有する。その人が、精神障害のために、特定の行動や決定を行うことができない場合には、彼らに作用するすべての人は、成年後見法（2000）の原則と規定を考慮しなければならない。この原則は、障害者権利条約の第12条に記述されており、法的行為能力に関連する国際標準の源泉になっている。総括すると、能力を欠如した認知症の本人を代理するいかなる介入も：
    - ・その人の利益でなければならない。
    - ・望ましい利益を確保する一方で、本人の自由制限は最小限でなければならない。
    - ・（コミュニケーションの適切な支援とともに）本人の過去および現在の希望を配慮しなければならない。
    - ・関係する他者の視点を配慮しなければならない。
    - ・彼らにある能力を活用し、新たなスキルが開発されるように、彼らを援助しなければならない。

2009年に英国のスコットランドで作成された「認知症の人々とケアラーのための権利章典」より（粟田主一：Dementia Friendly Communityの理念と世界の動き．老年精神医学雑誌28：458-465, 2017参照）

コーディネーションとネットワーキングの役割を担う社会資源

●地域包括支援センター

地域住民の保健医療の向上及び福祉の増進を支援することを目的に、心身の健康保持と生活の安定に必要な援助を包括的に行う地域の中核的機関。地域住民の多様な相談に応じ、①介護予防ケアマネジメント、②総合相談支援業務、③権利擁護業務、④包括的・継続的ケアマネジメント支援業務を通して、コーディネーションとネットワーキングを実践的に行っている。地域包括支援センターの所在は下記のURLを参照。
http://www.fukushihoken.metro.tokyo.jp/kourei/ninchi/gyakutai_madoguchi/soudan_madoguchi_hokatu.files/soudan_madoguchi_houkatu.pdf

●認知症支援コーディネーター

東京都では、保健師等の医療職を区市町村の地域包括支援センター等に配置し、認知症の疑いのある人の早期把握に努めるとともに、訪問による支援を行い、介護事業所、かかりつけ医、認知症サポート医、認知症疾患医療センター等と連携して、地域における認知症対応力の向上を図っている。東京都では、それぞれの区市町村に配置されている認知症地域支援推進員と協力して、コーディネーションとネットワーキングの役割を果たすことが期待されている。

●認知症地域支援推進員

認知症になっても、できる限り住み慣れた地域で生活を継続することができるように、医療機関、介護サービス、地域支援機関等の社会資源のコーディネーションとネットワーキングの役割を担う専門職。国の認知症施策において、区市町村・地域包括支援センター等に配置することとされている。東京都では、それぞれの区市町村において、認知症支援コーディネーターと役割分担を行いながら、コーディネーションとネットワーキングを実践することが期待されている。

●認知症サポート医

国が実施する認知症サポート医養成研修を受講した医師。認知症の地域連携体制の構築において中核的な役割を担い、かかりつけ医の相談に応じ、かかりつけ医の研修を企画し、医師会と地域包括支援センターとの連携を推進する。認知症初期集中支援チームの一員でもある。東京都内の認知症サポート医については以下のURLを参照。
http://www.fukushihoken.metro.tokyo.jp/zaishien/ninchishou_navi/soudan/iryou_kikan/support_meibo/index.html

●認知症初期集中支援チーム

認知症サポート医、認知症支援コーディネーター、認知症地域支援推進員等の専門職で構成される多職種協働チーム。認知症になっても本人の意思が尊重され、できる限り住み慣れた地域のよい環境で暮らし続けることができるように、相談応需、情報収集、アセスメント、家庭訪問、チーム員会議、かかりつけ医との連携等によって必要な支援を調整している。国の認知症施策の一環で、すべての区市町村に配置されている。

●居宅介護支援事業所

自宅で介護を受けている要介護者（要介護１～５の認定を受けた人）が、適切にサービスを利用できるように支援する事業所。担当する介護支援専門員（ケアマネジャー）が、ケアプランの作成・運用を支援し、介護全般の相談に応じる。東京都における居宅介護支援事業所一覧は下記を参照。
http://www.fukushihoken.metro.tokyo.jp/kourei/hoken/kaigo_lib/jigyo/shitei/togetsu.html

かかりつけ医と専門医療機関

●かかりつけ医

人々が暮らす身近な場所で、保健・医療に関するさまざまな相談に応じ、必要に応じて専門医療機関とも連携しながら認知症の診断や初期対応を行い、家族や地域とのつながりの中で、長期にわたるパートナーシップを築き、日常的な健康管理など継続的な医療を提供する医療機関。東京都では、認知症疾患医療センターがかかりつけ医認知症研修を実施している。

●認知症疾患医療センター

認知症に関する専門相談、鑑別診断、初期対応、急性期対応などの専門医療を提供するとともに、認知症の専門職を育成し、連携協議会を開催することなどによって地域連携体制の推進を図る専門医療機関。東京都内の認知症疾患医療センターについては下記のURLを参照。
http://www.fukushihoken.metro.tokyo.jp/zaishien/ninchishou_navi/torikumi/iryoucenter/index.html

● **もの忘れ外来、メモリークリニック**

認知症疾患医療センター以外にも、認知症の鑑別診断等の専門医療を提供している医療機関は多い。東京都では、下記のURLから認知症の診療に対応している最寄りの医療機関（病院、診療所）のリストを入手することができる。
http：//www.himawari.metro.tokyo.jp/qq13/qqport/tomintop/hospital/fk9023.php

生活支援、家族支援、経済支援、権利擁護支援に関係する社会資源

● **認知症サポーター**

認知症に対する正しい知識と理解を持ち、地域で認知症の人やその家族に対してできる範囲で手助けする認知症サポーター養成講座の受講者。認知症サポーター養成講座は、認知症にやさしい地域づくりを目標に、地域住民、金融機関やスーパーマーケットの従業員、小中学校の生徒などを対象に全国で実施されている。国の認知症施策の一環で、区市町村事業として実施されている。

● **認知症カフェ、ケアラーズカフェ**

認知症の本人や家族が気軽に過ごし、地域の人や専門家と相互に情報を共有することができる居場所。お互いを理解し合うとともに、地域づくりの拠点とされている。ケアラーズカフェは、ケアラー（家族の介護に携わる人）のためのカフェであり、情報交換や気軽なコミュニケーションを通して、ケアラーをサポートすることを目的としている。下記のURLを参照。
認知症カフェ ➡ http://ninchishocafe.jugem.jp/
ケアラーズカフェ ➡ http://cafearajin.com/

● **生活支援コーディネーター（地域支え合い推進員）**

介護予防・日常生活支援総合事業（総合事業）は、市町村が中心となって、地域の実情に応じて住民等の多様な主体が参画し、多様なサービスを充実させることで、地域の支え合い体制づくりを推進し、要支援者等に対する効果的・効率的な支援の実現を目指すものである。生活支援コーディネーターは、生活支援・介護予防サービスの体制整備を進めていくために、多様な主体による多様な取り組みのコーディネート機能（①資源開発、②ネットワーク構築、③ニーズと取組のマッチング）を担うものとされている。区市町村域（第1層）及び日常生活圏域（第2層）に配置される。下記のURLを参照。
http://www.mhlw.go.jp/file/06-Seisakujouhou-12300000-Roukenkyoku/0000192996.pdf

● **民生委員・児童委員**

民生委員は、厚生労働大臣から委嘱され、それぞれの地域において常に住民の立場に立って相談に応じ、必要な援助を行い、社会福祉の増進に努める人とされている。児童委員（地域の子供たちが元気に安心して暮らせるように、子どもたちを見守り、子育ての不安や妊娠中の心配ごとなどの相談・支援等を行う）を兼ねる。下記のURLを参照。
http://www.mhlw.go.jp/stf/seisakunitsuite/bunya/hukushi_kaigo/seikatsuhogo/minseiiin/index.html

● **認知症の人と家族の会**

「つどい」（介護家族のつどい、本人・若年のつどい、男性介護者のつどい）、「電話相談」、「会報の発行」などを通して、認知症の人と家族を支援する全国組織。各都道府県に支部があり、それぞれの地域で「つどい」が開催されている。下記のURLを参照。
http://aaj-tokyo.txt-nifty.com/

● **福祉事務所**

社会福祉法第14条に規定されている「福祉に関する事務所」。福祉六法（生活保護法、児童福祉法、母子及び寡婦福祉法、老人福祉法、身体障害者福祉及び知的障害者福祉法）に定める援護、育成または更生の措置に関する事務を司る。生活保護の申請は区市町村の福祉事務所で取り扱っている。以下のURLを参照。
http://www.mhlw.go.jp/stf/seisakunitsuite/bunya/hukushi_kaigo/seikatsuhogo/fukusijimusyo/index.html

● **社会福祉協議会**

住民が主体となって地域における福祉の問題を解決し、その改善向上を図るため、公私関係者の協力を得て地域福祉を推進することを目的とする民間団体。①地域住民の理解・関心を深め、住民参加を進めるための普及及び宣伝、②社会福祉関係機関・団体などの行う福祉活動の連絡調整及び助成、③社会福祉関連事業の調査・研究、④ボランティア活動の推進、⑤在宅福祉サービスの提供や各種相談事業、心身障害・高齢者・児童・母子福祉に関する事業、⑥各種援護資金、生活福祉資金の貸付、⑦協働募金への協力、歳末たすけあい運動などを行っている。「日常生活自立支援事業」の実施主体は社会福祉協議会である。区市町村社会福祉協議会の所在は以下のURLを参照。
http://www.fukunavi.or.jp/fukunavi/controller?cmd=lst&actionID=conslt&SVCSBR_CD=121

● **成年後見制度推進機関**

東京都では、各区市に設置された「成年後見制度推進機関」の運営を支援し、成年後見制度の普及促進を図っている。成年後見制度推進機関では、「成年後見制度について知りたい」「申し立ての手続きが分からない」「後見人になってくれる人を探している」など、制度全般に関するさまざまな相談への対応や、後見業務に関する研修を行っている。各区市の成年後見制度推進機関は下記のURLを参照。

http://www.fukushihoken.metro.tokyo.jp/kiban/sodan/kouken/torikumi.html

● **高齢者虐待に関する通報・相談窓口**

高齢者虐待の被害にあったり、虐待を発見したときの連絡先は、地域包括支援センターまたは区市町村の担当窓口である。下記のURLを参照。

http://www.fukushihoken.metro.tokyo.jp/kourei/ninchi/gyakutai_madoguchi/index.html

● **東京都消費生活総合センター**

東京都では、都内在住・在勤・在学者を対象に消費生活全般の相談に応じている。高齢者の経済被害等について本人・家族が相談する場合には「高齢者被害110番」(03-3235-3366)、介護事業者・民生委員など高齢者に身近にいる人が通報・問い合わせをする場合には「高齢者消費者見守りホットライン」(03-3235-1334)を利用する。下記のURLを参照。

https://www.shouhiseikatu.metro.tokyo.jp/sodan/sodan.htmlhttp://www.fukushihoken.metro.tokyo.jp/kiban/sodan/kouken/torikumi.html

若年性認知症に関係する社会資源

● **若年性認知症総合支援センター**

若年性認知症支援コーディネーターが、本人や家族からの多岐にわたる相談に対し、ワンストップで対応する。地域包括支援センターや医療機関等の専門機関からの相談についても、必要な助言を行い、相互に連携しながら若年性認知症の人への支援を行う。「東京都若年性認知症総合支援センター（相談専用電話番号03-3713-8205、平日9：00～17：00）」と「東京都多摩若年性認知症総合支援センター（相談専用電話番号042-843-2198、平日9：00～17：00）」がある。下記のURLを参照。

http://www.fukushihoken.metro.tokyo.jp/zaishien/ninchishou_navi/soudan/jakunen_center/index.html

● **若年性認知症コールセンター**

若年性認知症に関する多様な電話相談に応じる窓口。愛知県の認知症介護研究・研修大府センターに設置されている。相談専用電話は0800-100-2727（月～土10：00～15：00）。下記のURLを参照。

http://www.y-ninchisyotel.net/index.html

国や都の認知症関連施策に関する情報

● **東京都の認知症施策全般については下記のURLを参照**

http://www.fukushihoken.metro.tokyo.jp/zaishien/ninchishou_navi/

● **厚生労働省の認知症施策については下記のURLを参照。**

http://www.mhlw.go.jp/stf/seisakunitsuite/bunya/hukushi_kaigo/kaigo_koureisha/ninchi/index.html

● **介護予防・日常生活支援総合事業（総合事業）については下記のURLを参照。**

http://www.mhlw.go.jp/stf/seisakunitsuite/bunya/0000192992.html
